# A transient *in planta* editing assay identifies specific binding of the splicing regulator PTB as a prerequisite for cassette exon inclusion

**DOI:** 10.1007/s11103-024-01414-3

**Published:** 2024-03-05

**Authors:** Jorinde Loeser, Julia Bauer, Kim Janßen, Kevin Rockenbach, Andreas Wachter

**Affiliations:** Institute for Molecular Physiology (imP), University of Mainz, Hanns-Dieter-Hüsch-Weg 17, 55128 Mainz, Germany

**Keywords:** Editing, TRIBE, ADAR, Splicing, PTB, Polypyrimidine tract binding protein

## Abstract

The dynamic interaction of RNA-binding proteins (RBPs) with their target RNAs contributes to the diversity of ribonucleoprotein (RNP) complexes that are involved in a myriad of biological processes. Identifying the RNP components at high resolution and defining their interactions are key to understanding their regulation and function. Expressing fusions between an RBP of interest and an RNA editing enzyme can result in nucleobase changes in target RNAs, representing a recent addition to experimental approaches for profiling RBP/RNA interactions. Here, we have used the MS2 protein/RNA interaction to test four RNA editing proteins for their suitability to detect target RNAs of RBPs *in planta*. We have established a transient test system for fast and simple quantification of editing events and identified the hyperactive version of the catalytic domain of an adenosine deaminase (hADARcd) as the most suitable editing enzyme. Examining fusions between homologs of polypyrimidine tract binding proteins (PTBs) from *Arabidopsis thaliana* and hADARcd allowed determining target RNAs with high sensitivity and specificity. Moreover, almost complete editing of a splicing intermediate provided insight into the order of splicing reactions and PTB dependency of this particular splicing event. Addition of sequences for nuclear localisation of the fusion protein increased the editing efficiency, highlighting this approach’s potential to identify RBP targets in a compartment-specific manner. Our studies have established the editing-based analysis of interactions between RBPs and their RNA targets in a fast and straightforward assay, offering a new system to study the intricate composition and functions of plant RNPs in vivo.

**Supplementary Information:**

The online version contains supplementary material available at 10.1007/s11103-024-01414-3.

## Key message

We have established a fast and straightforward editing-based assay to analyse RBP/RNA interactions, demonstrating PTB binding to an intronic sequence adjacent to a cassette exon as prerequisite for its inclusion.

**Supplementary Information:**

The online version contains supplementary material available at 10.1007/s11103-024-01414-3.

## Introduction

The manifold functions of the different types of RNA classes are tightly linked to the RNA-binding proteins (RBPs) associated with them, forming highly dynamic and complex ribonucleoprotein particles (RNPs) that are responsible for executing diverse activities from the cellular to the organismal level. Several hundred proteins with annotated RNA-binding domains exist in the model plant *Arabidopsis thaliana* (Silverman et al. 2013). These but also other proteins lacking such conserved domains may form at least under certain conditions RNPs. Their detailed analysis and description as a basis for their functional characterisation remains a challenging task. The developments in high-throughput sequencing techniques now allows transcriptome analyses at unprecedented depth and width, resulting in highly resolved transcriptomes, e.g., for *A. thaliana* (Zhang et al. 2022). Among various co- and posttranscriptional RNA processing steps, alternative splicing (AS) of precursor mRNAs has been identified as a major source of transcriptome complexity and diversity in plants and other higher eukaryotes (Reddy et al. 2013; Staiger and Brown 2013).

In contrast to this massive expansion in our knowledge of transcriptome profiles in plants, slower progress has been made in elucidating the underlying mechanistic principles, i.e., which specific alterations in RNPs are responsible for the observed changes in transcript processing and regulation. RNA-seq studies of plant mutants with altered expression of RBPs provided evidence for their involvement in transcriptome regulation. For example, the analysis of *Arabidopsis thaliana* mutants that had either increased or diminished levels of polypyrimidine tract binding (PTB) proteins, which belong to the group of heterogenous ribonucleoprotein proteins (hnRNPs) (Wachter et al. 2012), revealed for 452 AS events reciprocal splicing shifts (Rühl et al. 2012), suggesting their identity as *bona fide* targets of PTB-mediated AS control. Transient co-expression of PTBs with splicing reporters based on the *PTB* pre-mRNAs, demonstrating auto- and cross-regulatory loops (Stauffer et al. 2010), or a mini-exon construct (Simpson et al. 2014) also substantiated their splicing-regulatory functions. However, these types of studies do not provide evidence for direct RBP/RNA interactions, and therefore alternative modes of actions cannot be excluded. Additional experiments such as electrophoretic mobility shift assays (EMSAs) can help to support the hypothesis of direct binding of an RBP to its proposed target RNA. Accordingly, recombinant PTB2 was shown to bind a part of the *PHYTOCHROME INTERACTING FACTOR 6* (*PIF6*) RNA in vitro, and combined with the AS shift observed for *PIF6* in *ptb* mutants, it was concluded that PTB2 can directly regulate this AS event in vivo (Rühl et al. 2012). Many other studies have examined the interaction between plant RBPs and RNAs in vitro (Reddy et al. 2013), e.g., demonstrating that UBP1 from *Nicotiana plumbaginifolia* is a uridylate-binding protein (Lambermon et al. 2000), examining the preference of AtRZ-1a binding to homoribopolymers (Kim et al. 2005), and investigating the sequence signature required for AtGRP7 binding via fluorescence correlation spectroscopy (Leder et al. 2014). However, major limitations of EMSA and related methods are their low-throughput, the requirement to generate and purify recombinant proteins, and that the assays are performed under in vitro conditions.

To overcome these limitations, significant effort has been made to develop and improve methods that allow profiling RNA/protein interactions from biological samples (Burjoski and Reddy 2021). On one hand, the RNA component can be captured via certain sequence or structural features followed by the identification of the associated proteins via mass spectrometry (Köster et al. 2017). On the other hand, an RBP of interest can be used to co-precipitate bound RNAs, also referred to as RNA immunoprecipitation (RIP) or, upon UV treatment to induce covalent bonds between RBPs and target RNAs in close vicinity, cross-linking immunoprecipitation (CLIP) (Hafner et al. 2021). Modified CLIP protocols such as iCLIP (individual-nucleotide resolution CLIP; König et al. 2010), even allow mapping the cross-linking site at nucleotide resolution. High-throughput sequencing of the immunoprecipitated RNAs (CLIP-seq) then allows a transcriptome-wide assessment of the binding regions of an RBP of interest. Accordingly, Zhang et al. (2015) identified via CLIP-seq the preference of the hnRNP HLP1 to bind A- and U-rich elements around polyadenylation sites and demonstrated this factor’s function in alternative polyadenylation in *A. thaliana*. Meyer et al. (2017) adapted iCLIP for the use in *A. thaliana* and observed that ~ 850 transcripts had crosslink sites for GRP7. By comparison with RIP-seq data, the authors identified ~ 450 high confidence targets detected by both methods, and observed an overrepresentation of U/C-rich motifs in the vicinity of GRP7’s crosslink sites.

The relatively high amounts of starting material required for CLIP and related methods represents a drawback in identifying the RNA targets of an RBP of interest, in particular if the analysis is to be restricted to low-abundant subpopulations of RNPs, e.g., from specific tissues, certain subcellular compartments, or in case of unstable processing intermediates. This constraint can be overcome using a more recently established genetic approach, in which an RBP of interest is expressed as a fusion protein with an RNA editing enzyme. The resulting editing sites are then determined via sequencing, which provides high sensitivity and therefore also works with low amounts of starting material. The first report of this type of approach was based on a study in *Drosophila melanogaster* from McMahon et al. (2016), who coined the term TRIBE (targets of RNA binding proteins identified by editing) for it. In their study, the catalytic domain of an adenosine deaminase (ADARcd) from *D. melanogaster* was fused to RBPs of interest. Adenosine deamination results in the formation of inosine, which is read as guanosine by cellular systems. Accordingly, an A-to-G conversion within an RNA corresponds to an editing event, which in turn suggests that an interaction of the corresponding RNA with the RBP of interest fused to the editing enzyme had taken place. A major limitation of the original TRIBE approach was its high rate of false negative results, i.e., many RBP/RNA interactions were not detected, due to a relatively low editing activity of the ADARcd. A follow-up study used a hyperactive version of ADARcd (hADARcd), containing the single E488Q amino acid exchange that was previously identified in a yeast screen of the catalytic domain from human ADAR2 for mutant proteins with increased editing efficiency and a reduced sequence preference at editing sites (Kuttan and Bass 2012). This HyperTRIBE (hTRIBE) approach resulted in the detection of far more significant editing sites compared to the study with the original ADARcd version (Rahman et al. 2018; Xu et al. 2018).

(h)TRIBE has an enormous potential as a complementary or alternative approach to methods based on RNP purification and can provide novel insight into RNP composition and functions (Xu et al. 2022). Recently, a combination of hTRIBE and iCLIP was applied to revisit the mRNA motifs bound by the N6-methyladenosine (m^6^A) reader protein ECT2 from *A. thaliana* (Arribas-Hernández et al. 2021a). Despite overall low editing proportions, several thousand editing sites could be identified and validated based on the comparison of the results for aerial and root tissues, hADARcd fusions with ECT2 and its homolog ECT3, and due to the overlap with targets identified by iCLIP or transcripts known to contain m^6^A modifications (Arribas-Hernández et al. 2021a, b). ECT2 is a cytosolic protein and evidence was provided that ECT2 and ECT3 can influence the abundance of their target RNAs via an unknown mechanism (Arribas-Hernández et al. 2021b). Numerous editing events were also reported upon transient expression of a fusion between the RBP UBP1c from *A. thaliana* and hADARcd in *N. benthamiana* leaves (Zhou et al. 2021). Interestingly, induction of effector-triggered immunity in the transiently transformed leaves resulted in a more than tenfold increase in the number of sites with altered nucleotide identity. Further studies will be needed to validate the targets via an independent approach and to examine the functional relevance of the proposed interactions. Very recently, Yin et al. (2023) reported editing-based identification of target RNAs in rice (*Oryza sativa*). As a proof that hTRIBE can also be applied in this species, the authors demonstrated for two different RBPs, each in fusion with hADARcd, editing of specific target RNAs in rice protoplasts. Moreover, stably transformed rice plants expressing a fusion between OsDRB1 (*O. sativa* Double-stranded RNA-Binding Protein 1) and hADARcd allowed editing-based detection of several hundred target RNAs. A large proportion of editing sites were located in non-coding regions of transcripts, and parallel analysis of leaf and root samples provided evidence for tissue-specific RBP/RNA interactions. These recent hTRIBE studies highlight this methods’ potential in the analysis of RBP/RNA interactions and also its general applicability in plants.

Besides ADAR, other types of editing enzymes such as members of the APOBEC (apolipoprotein B mRNA editing catalytic polypeptide-like) family catalysing cytosine-to-uracil editing exist (Salter et al. 2016). These proteins can be used in TRIBE-related approaches (Xu et al. 2022) to overcome limitations that are intrinsic to the ADAR enzyme, in particular that it can only edit adenosine residues and that it can result in a bias due to its sequence and structural preferences at editing sites (McMahon et al. 2016; Xu et al. 2018). Accordingly, the cytidine deaminase APOBEC1 was recently successfully used to discover targets of several RBPs via C-to-U conversion in human cell lines (Brannan et al. 2021). Similarly, a fusion of APOBEC1 to the m^6^A-binding YTH domain was used to profile m^6^A sites in mammalian cells, referred to as DART-seq (deamination adjacent to RNA modification targets; Meyer 2019). A study based on transient expression in *N. benthamiana* leaves provided first evidence that only hADARcd but not the other editing enzymes tested can result in specific base modifications (Zhou et al. 2021).

In this study, we have used the well-established interaction between the *MS2* hairpin RNA and the MS2 coat protein, both originally derived from the bacteriophage MS2 (Johansson et al. 1997; Lim and Peabody 1994), to test four different RNA editing enzymes for their ability to detect RNP formation upon transient expression in * Nicotiana benthamiana*. Upon identifying the hADARcd version with the E488Q mutation as the optimal choice, we expanded our study to the splicing regulatory proteins from the PTB family. We observed highly specific interaction between PTBs and a splicing reporter derived from the *PTB2* pre-mRNA, as supported by testing PTB homologs with different specificities and including a target RNA mutated in PTB binding motifs. Our findings indicate that the specificity of PTB homologs can already be defined at the step of RNA binding, and that this is a prerequisite for formation of the corresponding splicing variant. Based on their splicing regulatory activity, we demonstrate that the fusion proteins also retain the PTBs’ function in AS control. Furthermore, by using PTB-hADARcd fusions with nuclear localisation sequences, we provide evidence that this approach has the potential to identify compartment-specific RNPs. The here established RBP/RNA interaction assay, including the straightforward and quantitative readout via Sanger sequencing, provides a sensitive and fast test system to study the composition and functions of specific RNP complexes in living plant cells.

## Results

### *In planta* editing activity is observed for ADARcd, but not APOBEC proteins

**Fig. 1:**
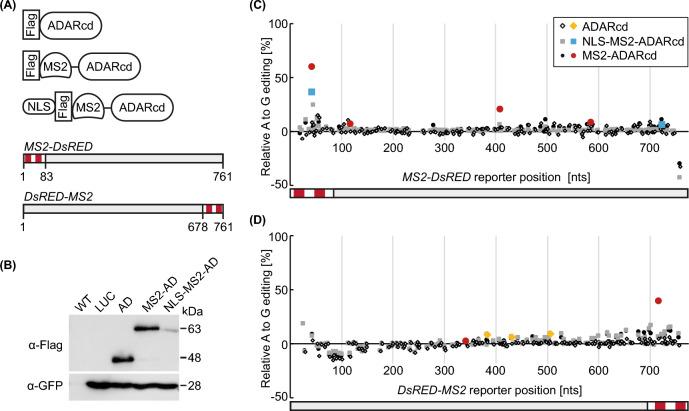
MS2-ADARcd causes editing of *MS2*-containing reporter RNAs. **A** Cartoons of the three constructs comprising the catalytic domain of ADAR (ADARcd), a Flag epitope tag, and for the third construct an NLS (nuclear localisation signal). The reporter constructs contain two *MS2* RNA stem loops (depicted in red) fused to the coding sequence of *DsRED*. The reporters are drawn to scale and numbers indicate nucleotide positions of the *MS2* and *DsRED* RNA regions. **B** Immunoblot analysis of samples from *N. benthamiana* leaves transiently transformed with ADARcd (AD), MS2-ADARcd, and NLS-MS2-ADARcd. Untransformed wild type (WT) and leaves transformed with Luciferase construct (LUC) serve as controls. GFP from co-transformation was detected as loading control. Samples were taken 2 days after infiltration. Positions of relevant size marker bands are indicated. **C**, **D** Quantitative analysis of A to G editing frequencies for all A bases along the reporter with the *MS2* RNAs located at the 5’ end **C** or 3’ end **D**. Samples were taken two days after infiltration and editing determined via Sanger sequencing of RT-PCR products. 3—4 biological replicates derived from two independent experiments, with significant changes depicted in colour. Further description of the data analysis is provided under Experimental procedures and applies to all corresponding displays in the other figures

To examine to which extent RNA editing enzymes derived from animals and fused to RBPs are functional *in planta*, we made use of the well-established interaction between the MS2 coat protein and the *MS2* hairpin RNA from the bacteriophage MS2 (Johansson et al. 1997; Lim and Peabody 1994). Accordingly, a reporter RNA containing the *MS2* region is expected to be bound and edited by the fusion protein. First, we tested the catalytic domain of ADAR (ADARcd), which is derived from *D. melanogaster* and was also used in the original TRIBE study (McMahon et al. 2016). To examine this protein’s activity *in planta*, we generated transformation constructs for expressing ADARcd either alone as a control or in fusion with the MS2 protein (Fig. 1A). A third construct additionally included the sequence encoding a nuclear localisation signal (NLS) to target the fusion protein and confine potential editing mainly to the nuclear compartment. The two reporter RNAs consisted of the coding sequence (cds) of *DsRED* and two copies of the *MS2* RNA hairpin positioned either up- or downstream of it (Fig. 1A). Immunoblot analysis upon transient transformation of *N. benthamiana* leaves resulted in strong signals for the ADARcd (predicted MW ~ 45.8 kDa) and MS2-ADARcd proteins (predicted MW ~ 60.1 kDa), whereas only a faint signal was detected in case of the NLS-containing version (predicted MW ~ 61.1 kDa; Fig. 1B). This weaker immunosignal may be a consequence of limited accessibility of the flag epitope tag used for detection, given that it is located downstream of the NLS in this construct. Alternatively, nuclear localisation of the fusion protein may cause poorer extractability or increased turnover.

To detect possible editing events, we analysed the sequences of the co-expressed reporter RNAs using Sanger sequencing of RT-PCR products. In case of editing at a particular position within the reporter, a relative increase in the guanosine to adenosine signal at this site is expected. We developed an analysis pipeline to quantify chromatogram signals and identified events with a significant increase in A-to-G editing upon expression of an ADARcd construct relative to a control transformed with a luciferase construct (details provided in Experimental Procedures and Supplementary Information, Data S1). As previously established for competitive PCR applications (Becker-André and Hahlbrock 1989), reverse transcription and PCR co-amplification were expected to maintain the starting ratio of the variants. Indeed, sequencing plasmids carrying single point mutations within the *DsRED* cds and mixed at different ratios confirmed that our analysis is sufficiently sensitive and reproducible to detect also events with a relatively low level of editing (Supplementary Information, Fig. S1). Analysing the *MS2-DsRED* reporter sequence identified four significant editing events upon MS2-ADARcd co-expression (red dots in Fig. 1C). Besides the number of editing positions, corresponding to the nucleobases in the target RNA that are accessible to the editing domain of the interacting fusion protein, this assay also provides a quantitative readout of editing strength based on the percentage of A to G conversions for each position. The strongest editing event was located within the *MS2* RNA region, where also binding of the fusion protein was expected. With an editing percentage above 50%, more than every second target RNA analysed here must have been bound and successfully edited by the MS2-ADARcd fusion protein. The same position was also significantly edited upon transformation with the NLS-MS2-ADARcd construct, which generated only one additional significant and weak editing event close to the 3’ end of the reporter (blue squares in Fig. 1C). Accordingly, overall weaker editing was seen for the fusion protein targeted to the nucleus, which may be explained by a lower protein abundance and/or a shorter time window for an interaction with and editing of the target RNA within the nuclear compartment. Given that the MS2 fusion protein is expected to be able to interact with the *MS2* RNA in the nucleus and cytosol, editing in this experiment may also occur in both compartments. Expressing ADARcd alone did not result in significant events for the *MS2-DsRED* reporter, indicating that the strong editing seen for the MS2-ADARcd proteins depends on the MS2 protein/RNA interaction. Editing occurred at fewer sites and to a weaker extent for the second reporter carrying the *MS2* RNAs at the 3’ end (Fig. 1D), with only one significant and pronounced event, again located within the *MS2* RNA region, upon co-expression of the MS2-ADARcd construct. The lower extent of editing may be explained by binding of the MS2 proteins at the 3’ end of this reporter, possibly resulting in limited accessibility of the RNA to the ADARcd domain. This reporter also resulted in several significant editing sites upon co-expression of ADARcd alone, however, the extent of editing was low at all of these sites (yellow rhomb symbols, Fig. 1D).

Next, we tested whether cytidine deaminases from the APOBEC family (Salter et al. 2016) could be an alternative to ADARcd to detect RBP-RNA interactions *in planta*. In contrast to ADAR, which in its natural context contains motifs for binding double-stranded RNA to associate with target RNAs, APOBEC proteins need to interact with an RBP to recognize their substrate RNA and perform editing. We first tested APOBEC1 from mouse expressed as a C- or an N-terminal fusion with the MS2 protein (Supplementary Information, Fig. S2a). Upon transient expression in *N. benthamiana* leaves, Flag-tagged MS2-APOBEC1 and APOBEC1-MS2 (predicted MW ~ 43.0 kDa) were detected as strong and weak immunosignals, respectively (Supplementary Information, Fig. S2B). Sequencing analysis of the co-expressed *MS2-DsRED* reporter did not reveal any significant editing event for both constructs (Supplementary Information, Fig. S2C), suggesting that the APOBEC1 fusion proteins are not functional under these conditions. As second member of the APOBEC family, we tested APOBEC3A from human. Upon cloning of an MS2-APOBEC3A fusion construct, several attempts of transforming this vector into *Agrobacterium tumefaciens* failed. This may be explained by cytotoxicity of the MS2-APOBEC3A protein expressed from the plant transformation vector in *A. tumefaciens*. To prevent protein generation in bacterial cells, we generated constructs containing intronic sequences within the *APOBEC3A* cds that need to be spliced in a eukaryotic cell to enable translation into a protein (Supplementary Information, Fig. S3A). Two different introns (Int_Cat_ and Int_PIV2*_), which were previously successfully used to control reporter gene expression in plant transformation experiments (Cazzonelli and Velten 2003; Luke Mankin et al. 1997), were inserted at two different positions each. Upon transient expression in *N. benthamiana* leaves, three out of the four different constructs resulted in an immunosignal at the expected position according to a predicted MW of ~ 38.5 kDa (Supplementary Information, Fig. S3B). We validated correct splicing of the inserted introns by directly sequencing RT-PCR products covering the respective exon-exon border regions of the transcripts from the *APOBEC3A* constructs. Correct splicing of Int_PIV2*_ at both positions was confirmed, whereas in case of Int_Cat_ usage of an upstream positioned alternative 3’ splice site resulted in the incorporation of the last 6 nt from the intron into the mature mRNA as part of the cds for both construct versions (Supplementary Information, Fig. S3C). Upon establishing APOBEC3A expression in a plant system, we investigated possible editing of the *MS2-DsRED* reporter upon co-transformation with the four different *APOBEC3A* constructs (Supplementary Information, Fig. S3D). As in case of APOBEC1, we did not find significant editing for any of the four constructs, indicating that also APOBEC3A is not a suitable editing enzyme in our experimental setup.

### The hyperactive hADARcd version results in stronger editing than ADARcd in transiently transformed *N. benthamiana* leaves

**Fig. 2:**
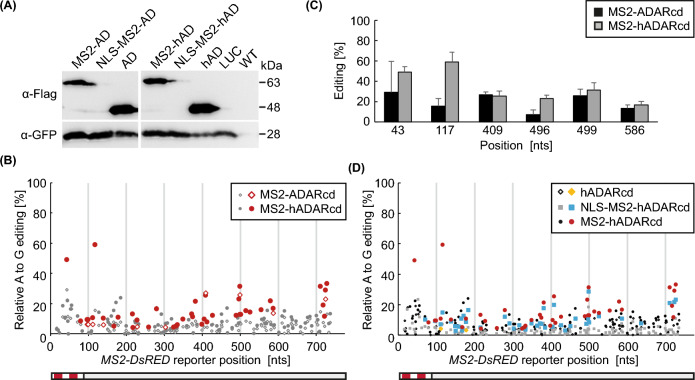
MS2-hADARcd results in stronger editing than MS2-ADARcd. **A** Immunoblot analysis of samples from *N. benthamiana* leaves transiently transformed with MS2-ADARcd, NLS-MS2-ADARcd, ADARcd, MS2-hADARcd, NLS-MS2-hADARcd, and hADARcd. Leaves transformed with Luciferase construct (LUC) and untransformed wild type (WT) serve as controls. GFP from co-transformation was detected as loading control. Samples were taken 3 days after infiltration. Total protein loaded per lane was 10 µg for (h)ADARcd and 20 µg for the other samples. Positions of relevant size marker bands are indicated. **B**, **C** Quantitative analysis of A to G editing frequencies for the A bases along the *MS2-DsRED* reporter, transiently transformed with MS2-ADARcd or MS2-hADARcd. Samples were taken three days after infiltration and editing determined via Sanger sequencing of RT-PCR products. With editing shown along the whole reporter RNA (B) and the extent of editing highlighted at six selected positions (C) along with standard deviation of the corrected editing quantity. Data derived from four biological replicates, with significant changes depicted in colour (B). **D** Quantitative analysis of A to G editing frequencies for all A bases along the *MS2-DsRED* reporter, transiently transformed with hADARcd, MS2-hADARcd, and NLS-MS2-hADARcd. Samples were taken three days after infiltration and editing determined via Sanger sequencing of RT-PCR products. Data are derived from four biological replicates, with significant changes depicted in colour. MS2-hADARcd data are the same as shown in (B) and are included here for a direct comparison

Based on the previous report of an improved performance of the hyperactive version of ADARcd (hADARcd) in TRIBE experiments in *D. melanogaster* (Xu et al. 2018), we performed a direct comparison of ADARcd and hADARcd using fusions with MS2 that were co-expressed with the *MS2-DsRED* reporter in *N. benthamiana*. Immunoblot analysis revealed strong accumulation of hADARcd and MS2-hADARcd proteins, with no visible effect of the E488Q mutation on protein accumulation (Fig. 2A). As seen before, addition of an NLS resulted in strongly reduced immunosignals. The editing analysis of the *MS2-DsRED* reporter revealed far more significant sites for MS2-hADARcd (41 positions) compared to MS2-ADARcd (10 positions; Fig. 2B), being in line with the results from the previous study in *D. melanogaster*. This analysis was performed 3 d after leaf infiltration, providing an explanation for the higher number of significant editing sites for MS2-ADARcd compared to the editing outcome 2 d after infiltration shown in Fig. 1. A direct comparison of the editing extent at six sites with significant changes in case of MS2-hADARcd showed that these adenosines can also be edited by MS2-ADARcd, however, sometimes at lower level or with higher variation and therefore not fulfilling our significance criteria (Fig. 2C). Next, we tested whether the increased editing efficiency of hADARcd is also linked to a higher background level. Compared to 41 events edited by MS2-hADARcd on the *MS2-DsRED* reporter, only two significant events were detected in case of the hADARcd protein alone (Fig. 2D). Both of these events showed only minor editing proportions. The NLS-MS2-hADARcd version also resulted in significant editing at many sites (43 positions; Fig. 2D); however, in most cases a higher extent of editing was seen for the NLS-free version.

Having identified hADARcd as the most promising candidate for our approach, we further characterized reporter editing in the *N. benthamiana* system. Analysing samples from two to five days after infiltration showed that MS2-hADARcd protein levels and the relative editing are highest at the time points 2 d and 3 d (Supplementary Information, Fig. S4). Accordingly, these early time points were chosen for the subsequent analyses. We also further analysed the specificity of editing observed with the MS2 system. First, we compared MS2-hADARcd-mediated editing of the *MS2-DsRED* reporter and a *DsRED* RNA without the *MS2* hairpins (Supplementary Information, Fig. S5A, B). Many editing sites were found for both RNAs; however, the two strongest editing events were unique to the *MS2-DsRED* reporter (Supplementary Information, Fig. S5B). Given that hADARcd alone results in only few and weak significant editing sites for the *MS2-DsRED* reporter (Fig. 2D), we concluded that most of the background seen here is probably a result of promiscuous RNA binding through the MS2 part of the MS2-hADARcd protein. We also extended the editing analysis to a co-expressed unrelated *GFP* RNA that does not contain the *MS2* stem loops. Again, more significant editing events and overall stronger editing was detected for MS2-hADARcd compared to hADARcd (Supplementary Information, Fig. S5C), further supporting that the MS2 part is largely responsible for background editing of the fusion protein. Interestingly, more editing of the *GFP* RNA was seen when MS2-hADARcd was co-expressed with *DsRED* RNA compared to co-expression with its authentic binding target *MS2-DsRED* (Supplementary Information, Fig. S5D). Accordingly, the absence of the *MS2* target RNA seems to enhance unspecific binding of MS2-hADAR to other RNAs, such as the *GFP* RNA here. Previous studies demonstrated ADARcd’s preference to edit unpaired adenosines embedded within a structured RNA region (McMahon et al. 2016; Xu et al. 2018). However, comparing structural features and editing along the *MS2-DsRED* reporter did not reveal any correlation in our analysis (Supplementary Information, Fig. S6).

### PTB2-hADARcd specifically edits its target RNA, establishing PTB2 binding as a prerequisite for formation of the corresponding AS variant

**Fig. 3:**
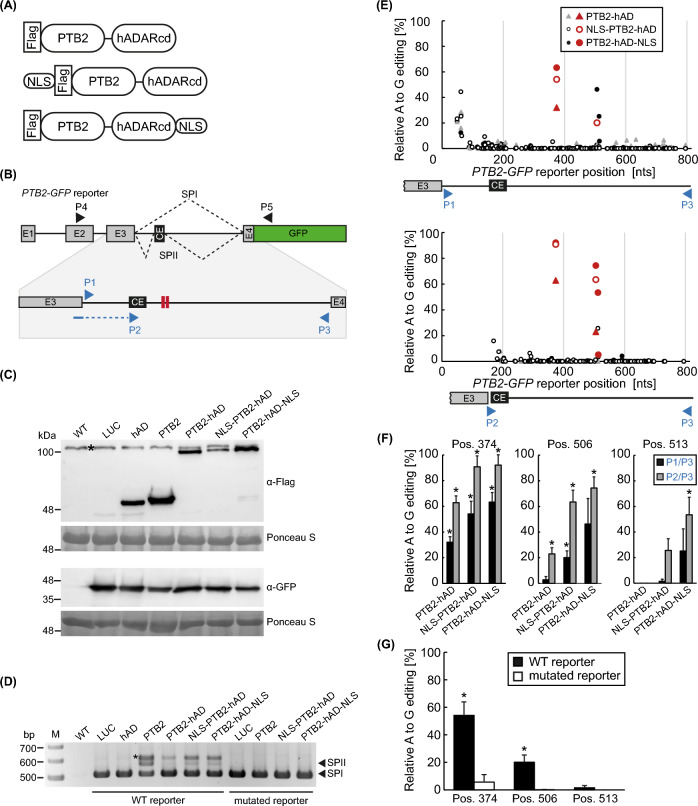
PTB2-hADARcd shows specific editing of *PTB2*-based reporter RNA. **A** Construct cartoons of AtPTB2 fusions with hADARcd, containing a Flag epitope tag and either no, an N- or a C-terminal NLS. **B** Cartoon of a splicing reporter containing the 5’ genomic region of *AtPTB2* fused to the cds of *GFP* (Stauffer et al. 2010). The area between exon E3 and E4 is shown enlarged below, depicting the binding sites of primers P1—P3 (blue arrows) and PTB2 binding motifs (red). Primers P4/P5 were used for co-amplification PCR shown in (D). Reporter is drawn to scale. Reporter positions in the following displays include intronic and exonic sequences, with the first intronic position downstream of exon 3 being defined as position 1. SPI and SPII refer to the splicing variant without and with the cassette exon (CE), respectively. **C** Immunoblot analysis of samples from *N. benthamiana* leaves transiently transformed with indicated constructs (hADARcd abbreviated as hAD) and co-infiltrated with the *PTB2-GFP* splicing reporter in its WT sequence. Leaves transformed with Luciferase construct (LUC) and untransformed wild type (WT) serve as controls. The sample set was probed on two blots with antibodies against Flag and GFP, respectively, and Ponceau S staining serving as loading control. Samples were taken 3 days after infiltration. Positions of relevant size marker bands are indicated. Band marked with an asterisk corresponds to a background signal that is also present in the WT sample. **D** Co-amplification PCR on the *PTB2-GFP* splicing reporter upon co-expression of controls (LUC, hAD) or indicated PTB2 constructs, detecting the two major splice variants SPI (512 nts) and SPII (570 nts). Band marked with asterisk does not correspond to any known/detectable splicing variant and, based on its absence in Bioanalyzer runs, may represent a gel running artifact. The PCR products were separated on an agarose gel and visualized via ethidium bromide staining. M, size marker. **E**–**F** Quantitative analysis of A to G editing frequencies for the A bases along the region flanking the cassette exon of the reporter, transiently transformed with PTB2-hADARcd, NLS-PTB2-hADARcd, or PTB2-hADARcd-NLS. Two intermediate splice versions were sequenced with one containing both intronic regions surrounding the cassette exon (P1/P3 primer combination) and the other having the first intron already spliced out (P2/P3 primer combination). Significant changes are depicted in colour (E) and the extent of editing is separately shown for three positions (F; asterisks indicate significant events) along with standard deviation of the corrected editing quantity. Aligned reporter region indicates approximate positions of editing in intron/exon context. Samples were taken three days after infiltration and editing determined via Sanger sequencing of RT-PCR products. Data derived from 4 to 7 biological replicates. **G** Quantitative analysis of A to G editing frequencies for the A bases of the WT or mutant reporter upon transient co-expression with NLS-PTB2-hADARcd. The intermediate splice version containing both introns flanking the cassette exon (P1/P3 primer combination) was sequenced, and the extent of editing is shown for three positions (asterisks indicate significant events). Samples were taken three days after infiltration and editing determined via Sanger sequencing of RT-PCR products. Data derived from 8 biological replicates

Having established an editing-based RBP/RNA in vivo interaction assay, we extended our experiments to the PTB proteins from *A. thaliana* that function as important regulators of AS events (Rühl et al. 2012; Simpson et al. 2014; Stauffer et al. 2010). PTBs were previously identified as components of the mRNA-binding proteome (Reichel et al. 2016), however, no evidence for their direct and in vivo binding to specific target RNAs was reported so far. To address this aspect, we generated a set of PTB2-hADARcd fusion constructs for hTRIBE studies (Fig. 3A). Given the previous report of PTB2 localization in different compartments (Stauffer et al. 2010), and that fusing this protein to hADARcd may affect its subcellular targeting, we also generated variants with NLS-encoding sequences, either containing an SV40 and bipartite NLS, respectively, at the N- or C-terminus of the fusion proteins (Fig. 3A). The presence of this additional NLS is expected to enhance the efficiency of targeting the fusion protein to the nucleus, in which AS regulation is taking place. As a potential substrate for editing in these transient assays, we co-expressed a splicing reporter consisting of the *AtPTB2* pre-mRNA sequence from the 5’ end of the transcript to a region within exon 4, which is positioned downstream of the alternatively spliced cassette exon, and fused to the cds of *GFP* (Fig. 3B). This *PTB2-GFP* reporter was previously used to demonstrate PTB-dependent auto- and cross-regulation (Stauffer et al. 2010), where elevated PTB levels trigger splicing to the unproductive SPII variant at the expense of the protein-coding SPI transcript. These previous findings indicated that PTBs bind their own pre-mRNAs to control the splicing outcome.

Transient expression in *N. benthamiana* followed by immunoblot analysis confirmed the formation of the control proteins hADARcd and PTB2 (predicted MW ~ 48 kDa) as well as the PTB2-hADARcd fusions without (predicted MW ~ 93.3 kDa) or with NLS (predicted MW ~ 94.3 kDa and ~ 95.6 kDa, respectively, for the fusion with the NLS at the N- and C-terminus; Fig. 3C). Detection of the reporter-derived GFP protein resulted in the weakest signal upon co-expression of the PTB2 protein alone (also see Fig. 5B), suggesting it causes the strongest negative feedback regulation. Accordingly, co-amplification of the reporter-derived splicing variants via RT-PCR confirmed a PTB-induced AS shift of the reporter (Fig. 3D). Furthermore, a mutant reporter lacking pyrimidine-rich motifs downstream of the cassette exon (Supplementary Information, Fig. S7a) resulted only in the SPI variant, probably due to impaired PTB binding. Based on the splicing ratios of the WT reporter, the strongest splicing shift was induced by PTB2 alone followed by the two NLS-containing fusions. Accordingly, fusion with the ADARcd domain diminished but did not abolish the splicing regulatory activity of PTB2, and enhanced splicing activity was seen in the presence of an NLS, probably due to a more efficient nuclear targeting.

Having proven the expression and splicing regulatory activity of the PTB2-hADARcd fusion proteins, we next examined whether specific editing of the reporter sequence can be detected as well. Given that PTB binding must already occur before intron removal and that the edited positions may lie within the introns, we focused in our editing analysis on transcripts that are not yet fully processed. To this end, we used random hexamer primers for cDNA synthesis, thereby covering also pre-mRNAs such as non-polyadenylated and nascent transcripts. Furthermore, at least one of the primers in the following PCR reaction was binding to an intronic region (Fig. 3B). The first RT-PCR fragment to be analysed was generated with primers P1 and P3, binding at the beginning of the upstream intron and at the end of the downstream intron, respectively, relative to the PTB-dependent cassette exon (Fig. 3B, E). In this fragment, the position A374 (counting relative to the first nucleotide in the intron downstream of exon 3) was significantly edited by all three PTB2-hADARcd versions. The extent of editing was higher for the NLS-containing versions, with more than 60% editing for the PTB2-hADARcd-NLS fusion. Downstream of this common editing site, one additional significant editing event was detected at position A506 for the NLS-PTB2-hADARcd samples. This site was showing an even higher editing ratio in presence of PTB2-hADARcd-NLS; however, it was not called significant in this case due to a higher variation between replicates and the stringent significance criteria. Given that the P1/P3 primer combination should detect all reporter RNAs unspliced in this region, including those being not bound by PTB2 and therefore expected to be spliced to SPI and not SPII, we analysed another RT-PCR product, derived from the second primer pair P2/P3 that is specific for intermediates being spliced to the PTB-dependent SPII variant including the cassette exon (Fig. 3B, E). This specificity was achieved by using the P2 primer, spanning the splicing-derived border between the upstream exon 3 and the cassette exon (Fig. 3B), i.e., all transcripts with the corresponding primer binding site must be spliced in the intron upstream of the cassette exon, which determines their splicing to SPII production. In line with this rationale, we found more and stronger editing for the P2/P3 fragment compared to the P1/P3 RT-PCR product (Fig. 3E, F). For the position A374 of the reporter, almost 100% editing was observed in case of the two NLS-containing PTB2-hADARcd constructs, indicating PTB2 binding is a prerequisite for splicing to SPII. At this and the two other significant editing positions, the NLS-free fusion showed weaker or even non-significant editing compared to the NLS-containing versions.

To examine the specificity of the editing, we then analysed the sequence of the P1/P3 fragment upon co-expressing NLS-PTB2-hADARcd with the mutated reporter lacking the PTB binding motifs (Fig. 3G). In contrast to the WT reporter, the mutant reporter showed no editing at positions A506 and A513, while editing at position A374 was strongly diminished and non-significant. Moreover, sequencing RT-PCR products of the reporter upon co-expression with PTB2 and hADARcd alone as well as a *DsRED* RNA co-expressed with hADARcd alone or PTB2-hADARcd fusions did not reveal any significant editing event for these controls (Supplementary Information, Fig. S7B-D), further highlighting the specificity of the editing caused by the PTB2-hADARcd proteins in the splicing reporter. This also supports our previous conclusion that most of the background editing seen for the MS2-hADARcd fusion results from promiscuous binding of the MS2 protein to various RNA regions rather than being caused by unspecific editing by hADARcd itself.

**Fig. 4:**
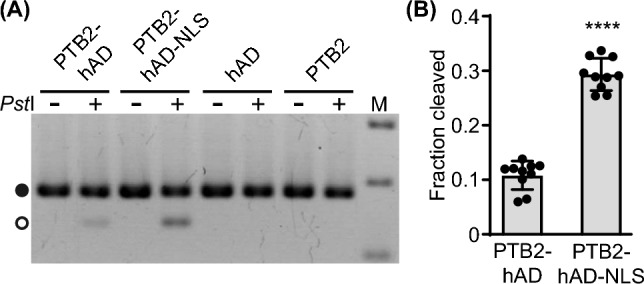
CAPS assay validates increased editing for NLS-containing PTB2-hADARcd protein. **A** Agarose gel picture of DNA samples without (“- “) or with (“ + ”) *Pst*I treatment for CAPS-based detection of editing at A374. DNA fragments were separated on an agarose gel and visualized via ethidium bromide staining. Black and white circle indicate uncleaved and cleaved fragment, respectively. Samples are derived from *N. benthamiana* leaves infiltrated with indicated constructs and *PTB2-GFP* splicing reporter as described before. **B** Quantitative analysis of fraction of cleaved DNA based on Bioanalyzer measurements. Data are derived from two independent experiments with a total of 10 replicates each shown as dots. Bars depict mean values; error bars correspond to standard deviation. Statistical significance was determined by unpaired, two-tailed t-test (P value: ****P < 0.0001)

To validate the NLS-dependent increase in editing via an independent experimental approach, we developed an assay based on cleaved amplified polymorphic sequence (CAPS) to detect the extent of editing at the reporter position A374. A sequence-modified primer was designed that introduces a *Pst*I restriction site into RT-PCR products derived from reporter transcripts edited at A374 (Supplementary Information, Fig. S8). Accordingly, *Pst*I treatment of the RT-PCR products derived from edited RNAs is expected to result in cleavage and a size shift, where the fraction of the shifted band should correlate with the extent of editing. In agreement with this assumption, we were able to detect via CAPS editing of *PTB2-GFP* reporter transcripts by the PTB2-hADARcd proteins with and without NLS, whereas no cleavage was seen for the negative controls, namely the unfused hADARcd and PTB2 (Fig. 4A). Moreover, quantification of the cleaved fraction confirmed significantly stronger editing for the NLS-containing compared to the NLS-free PTB2-hADARcd version (Fig. 4B), verifying the findings from the sequencing-based analysis (Fig. 3).

### PTB selectivity in AS control is also reflected on the level of editing, indicating specific target binding

**Fig. 5:**
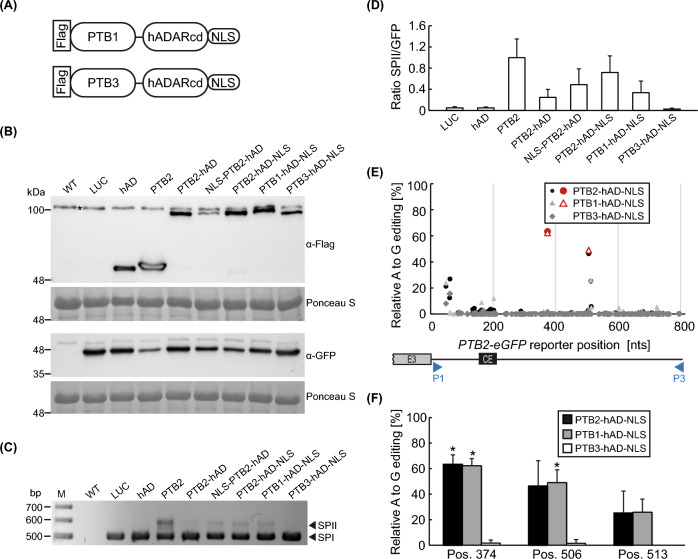
Specificity of PTB proteins is reflected by the editing outcome of a *PTB2*-based splicing reporter. **A** Cartoons of AtPTB1 and AtPTB3 fusions to hADARcd, containing an N-terminal Flag isotope tag and a C-terminal nuclear localization sequence (NLS). **B** Immunoblot analysis of samples from *N. benthamiana* leaves transiently transformed with control (LUC, hADARcd (hAD), PTB2) or PTB-hADARcd fusion constructs, and co-infiltrated with the *PTB2-GFP* splicing reporter. The sample set was probed on two blots with antibodies against Flag and GFP, respectively, and Ponceau S staining serving as loading control. Samples were taken 3 days after infiltration. Positions of relevant size marker bands are indicated. Band marked with an asterisk corresponds to a background signal that is also present in the WT sample. **C**, **D** Co-amplification PCR of splice variants SPI/SPII (C) and qPCR analysis of relative SPII levels (D) derived from *PTB2-GFP* reporter upon co-expression with different controls and PTB-hADARcd fusions. The PCR products shown in (C) were separated on an agarose gel and visualized via ethidium bromide staining. In (D), ratio for the PTB2-transformed sample was set to 1 and data are mean values from 6 to 8 biological replicates; error bars correspond to standard deviation. **E**–**F** Quantitative analysis of A to G editing frequencies for the A bases along the cassette exon flanking region of the reporter, that was transiently expressed with indicated PTB-hADARcd constructs. An intermediate splice version containing both introns surrounding the cassette exon (CE) was analysed using P1/P3 primers (further details described in Fig. 3). Significant changes are depicted in colour (E) and the extent of editing is highlighted at three relevant positions (F; asterisks indicate significant events) along with standard deviation of the corrected editing quantity. Samples were taken three days after infiltration and editing determined via Sanger sequencing of RT-PCR products. Data derived from 4 to 7 biological replicates

Previous analysis of PTB feedback regulation in *A. thaliana* revealed that the closely related PTB1 and PTB2 proteins cross-regulate each other via AS of their corresponding pre-mRNAs (Stauffer et al. 2010). This type of cross-regulation was not observed for the more distantly related PTB3, which was not able to induce an AS shift for the *PTB2*-based splicing reporter. To examine whether this specificity is also reflected on the level of PTB binding as detected via RNA editing, we expanded our analysis by generating constructs for the expression of PTB1-hADARcd-NLS and PTB3-hADARcd-NLS (Fig. 5A). Upon transient transformation of *N. benthamiana* leaves, the PTB1-hADARcd-NLS (predicted MW ~ 92.4 kDa) and PTB3-hADARcd-NLS (predicted MW ~ 97.0 kDa) proteins were detectable in immunoblots (Fig. 5B). Analysing the splicing pattern of the co-expressed *PTB2-GFP* splicing reporter confirmed for the hADARcd fusion proteins the specificity previously reported for the unfused PTBs: the PTB1 and PTB2 fusions altered reporter splicing with an increase in SPII, while PTB3-hADARcd-NLS was not able to induce splicing to the unproductive variant SPII (Fig. 5C, D). In line with this, significant editing of the reporter was seen for PTB1-hADARcd-NLS and PTB2-hADARcd-NLS, but not for PTB3-hADARcd-NLS (Fig. 5E, F). Accordingly, hTRIBE in this assay allows identifying the targets of RNA-binding proteins with high precision. Furthermore, these data suggest that specific binding of the *PTB2* pre-mRNA by PTB1 and PTB2 is a prerequisite for triggering the AS change to the unproductive SPII variant.

## Discussion

### Choice of editing enzyme for detecting RBP targets

Building on the well-known interaction between the MS2 protein and the *MS2* hairpin RNA (Johansson et al. 1997; Lim and Peabody 1994), we have explored in this study the potential of four different editing enzymes to detect RNA/RBP interactions *in planta*. Ten sites on an *MS2-DsRED* reporter were specifically edited by MS2-ADARcd, and using MS2-hADARcd resulted in a more than fourfold increase in the number of significant editing positions (Fig. 2). Accordingly, the catalytic activity of ADAR enzymes derived from *D. melanogaster* is preserved upon *in planta* expression and can result in multiple editing events on a target RNA. The increased number of significant editing sites as well as overall higher editing proportions in case of the hyperactive version hADARcd confirms its superior performance in (h)TRIBE applications and is in line with previous findings in *D. melanogaster* (Xu et al. 2018). Strikingly, the two strongest editing sites in the vicinity of the *MS2* RNA loops displayed A-to-G conversion rates of 50% and above in the presence of MS2-hADARcd, highlighting that robust editing activity can be achieved in plants. In line with our findings, editing percentages of 50% and higher at the most pronounced editing sites were observed for a target RNA with two internal *MS2* RNA loops that was co-expressed with MS2-hADARcd in rice protoplasts (Yin et al. 2023). In case of NLS-MS2-hADARcd, the number of significant editing sites was similar as in case of the NLS-free version, albeit editing rates were in general lower (Fig. 2D). The strongly diminished immunosignal for NLS-MS2-(h)ADARcd compared to MS2-(h)ADARcd may be explained by an increased turnover of the fusion proteins in the nucleus or impaired antibody recognition of the epitope tag in the corresponding fusion proteins due to the presence of the N-terminal NLS. Accordingly, the different editing rates observed for the NLS-free and NLS-containing fusions could reflect different protein abundance. Alternatively, restricting the potential interaction to the nuclear compartment may shorten the time window available for editing. A recent study in mouse fibroblasts used the MS2 system to compare the performance of hTRIBE and CLIP upon genetic tagging of the *β-actin* mRNA with a cluster of 24 *MS2* hairpin RNAs (Biswas et al. 2020). Both techniques identified the *MS2*-tagged *β-actin* mRNA as the top hit among MS2-interacting RNAs. Interestingly, the MS2-hTRIBE approach resulted in additional targets that did not contain *MS2*-related sequences or structures, but were in spatial proximity to the *β-actin* locus in the nucleus and may result from *trans*-editing.

Besides the two ADARcd versions, we also tested MS2 fusions with the cytidine deaminases APOBEC1 from mouse and APOBEC3A from human for their editing activity upon expression in *N. benthamiana* leaves. Neither of the two fusion proteins resulted in any reporter editing. As both types of fusion proteins were readily detectable via immunoblot analysis and given the sensitivity of our MS2-based test system, we conclude that the corresponding proteins don’t exhibit editing activity in our experimental setting. In contrast, rat APOBEC1 was successfully used in two studies with human cells that identified RNA targets based on C-to-U conversions. Accordingly, expressing a fusion between APOBEC1 and the m^6^A-binding YTH domain provided a novel approach to globally map m^6^A sites, referred to as DART-seq (Meyer 2019). Recently, Brannan et al. (2021) reported that STAMP (Surveying Targets by APOBEC-Mediated Profiling) successfully captured the RNA targets of three different RBPs in human cell lines. Transiently expressing fusions between the RBP AtUBP1c and several editing enzymes in *N. benthamiana* leaves resulted only in case of the hADARcd fusion in a large number of potential editing events (Zhou et al. 2021). For the alternative adenine deaminase TadA and three different known or predicted cytidine deaminases, the patterns of all possible nucleotide variations were similar to the control and therefore can be considered as background (Zhou et al. 2021). Interestingly, a fusion between human APOBEC3A and nCas9 was successfully used for cytidine deamination on the DNA level in three crop species (Zong et al. 2018), demonstrating that this editing enzyme in principle can be active in plants. While all of the editing enzymes successfully used so far in TRIBE-related approaches are derived from non-plant systems, RNA editing also occurs in plants with prevalent C-to-U editing in their organelles (Small et al. 2020). Thus, these organellar editing factors may provide novel candidates for TRIBE-related approaches in plants and possibly also other species. Akin to the identification of RBP targets via RNA editing, covalent modification of target RNAs by expressing an RBP of interest fused to a poly(U) polymerase was reported, referred to as RNA tagging (Lapointe et al. 2015). While this method was successfully used in *Saccharomyces cerevisiae*, it is unclear if it can also be established in multicellular eukaryotes. Furthermore, RNA tagging is restricted to modifications at the end of transcripts and the results may also be affected by RNA binding of the poly(U) polymerase.

### Editing outcome: specificity, sequence context, and extent of editing

We first used the MS2 system to examine how parameters such as the design of the editing reporter, the type of editing enzyme, and the time point of sampling after leaf infiltration affect the editing outcome. We consistently observed the strongest editing by MS2-(h)ADARcd at a position within or in close proximity to the two copies of the *MS2* loop present in the reporter. The editing frequency at these positions was typically around 50% or higher, meaning that at least every second transcript of the reporter had been bound and edited by the MS2 fusion protein. Editing was also observed at several other positions of the *MS2-DsRED* reporter, with a total of 41 significant editing sites for MS2-hADARcd. However, the extent of editing at these sites was lower compared to those nearby the *MS2* region. Most of the sites showed A-to-G conversion upon co-expression of MS2-hADARcd and MS2-ADARcd, but in case of the less active MS2-ADARcd fusion, many of the events showed weaker editing and therefore were not called significant. This observation is consistent with a comparison between ADARcd and hADARcd in the study from Xu et al. (2018). Importantly, expression of (h)ADARcd alone, i.e., not fused to any RBP, in *N. benthamiana* resulted in no editing or only very few significant events with low editing proportions. This observation is again consistent with findings in *D. melanogaster* (McMahon et al. 2016; Xu et al. 2018). Moreover, when co-expressing the MS2-hADARcd with a *DsRED* RNA construct lacking the *MS2* hairpins, we also detected multiple significant editing sites, several of which corresponded to the equivalent sites in the *MS2-DsRED* reporter. This finding points at promiscuous RNA binding caused by the MS2 protein. In line with this assumption, MS2-hADARcd also resulted in more background editing of a co-expressed *GFP* mRNA in comparison to the hADARcd construct, and this editing by MS2-hADARcd was diminished when the authentic binding target *MS2-DsRED* was co-expressed. Accordingly, our transient expression system allows analysing RBP/RNA interactions in a straightforward manner, including conditions where different RNAs can compete for binding. The strong editing seen here in the *MS2* region of the reporter is in line with the expectation that the MS2-(h)ADARcd proteins bind to the *MS2* hairpins and cause editing nearby. The additional weaker editing events can result from *trans*-editing depending on the RNA structural conformation or be the consequence of unspecific MS2 binding.

The transient expression system used in this study is expected to result in constitutive and strong expression of the RBP fusion and potential target RNAs, which may also result in unspecific interactions. However, our experimental series with PTB-hADARcd fusion proteins revealed that the editing outcome in this system can fully reflect the RBP’s authentic and specific binding behaviour. Accordingly, PTB2-hADARcd resulted in one major editing event at A374 that is positioned in the intron downstream of the PTB-regulated cassette exon of the splicing reporter (Fig. 3E). Compared to PTB2-hADARcd, the NLS-containing versions NLS-PTB2-hADARcd and PTB2-hADARcd-NLS caused even stronger editing at the very same position, in line with the fact that the interaction with the not yet fully spliced pre-mRNA of the reporter is taking place in the nucleus. Furthermore, when restricting the analysis to transcripts that are dedicated to splicing into the PTB2-induced AS variant including the cassette exon, more than 90% of the nucleotide signal at position A374 was converted from adenosine to guanosine upon co-expression of the NLS constructs. This clearly indicates that PTB2 binding is a prerequisite for inclusion of the cassette exon, as opposed to a model that a certain fraction of the pre-mRNA is constitutively spliced to the respective variant. The high extent of editing detected for the PTB interaction in the present study underlines that hTRIBE can be highly efficient in plants, also considering that the PTB2 interaction with the pre-mRNA has to be short-lived. Relatively high editing percentages at a large number of sites were also reported for rice plants expressing OsDRB1-hADARcd (Yin et al. 2023) and Hrp48-(h)TRIBE in *D. melanogaster* cells (Xu et al. 2018). In contrast, most of the events identified for the hADARcd fusions with the m^6^A-binding ECT2 and ECT3 proteins showed low editing proportions of a few percent or below in *A. thaliana* (Arribas-Hernández et al. 2021a, b). Such weak editing proportions may be explained by low expression or activity of specific hTRIBE fusion proteins, or be the consequence of a relatively weak or low abundant RNA/protein interaction.

Besides the high editing rates observed for the *PTB2* reporter RNA, we could demonstrate that the hTRIBE-based identification of PTB targets is highly specific. Accordingly, editing at A374 of the *PTB2* reporter and few other sites located downstream within the same intron, was completely lost when a mutated reporter lacking the PTB binding sites was used. The following findings further supported the high level of specificity of PTB2-hADARcd-mediated editing: i) hADARcd alone did not cause significant editing of the *PTB2* reporter pre-mRNA; ii) NLS-containing versions of PTB2-hADARcd caused stronger editing, indicating that editing takes place in the course of pre-mRNA splicing in the nucleus; and iii) PTB2-hADARcd did not cause any editing of a co-expressed *DsRED* RNA.

Previous studies have revealed widespread and overlapping functions of PTB1 and PTB2 from *A. thaliana* in AS regulation. In contrast, the more distantly related PTB3 did not cross-regulate splicing of a *PTB2*-based splicing reporter (Stauffer et al. 2010) and was not found to play a role in global AS control (Rühl et al. 2012). To investigate whether our editing system can reflect this specificity, we compared the editing activity of the three different PTB homologs, each fused with hADARcd-NLS, when co-expressed with the *PTB2*-based splicing reporter. In line with previous findings (Stauffer et al. 2010), only the PTB1- and PTB2-containing fusions triggered AS of the reporter to the variant containing the cassette exon, reflecting the reported cross- and autoregulatory mechanism, respectively. The PTB1- and PTB2-hADARcd-NLS fusions resulted in efficient and consistent reporter editing. In contrast, PTB3-hADARcd-NLS did not result in any significant editing event. On one hand, this clearly underlines the specificity of PTB-mediated editing in our system, which allows discriminating even between homologs from one family of splicing regulators. On the other hand, these findings suggest that the specificity of PTB proteins is already determined at the level of RNA binding. A correlation between RNA editing and splicing to the cassette exon inclusion variant of the reporter is not only seen in comparison of the three PTB homologs, but also when analysing the effect of adding an NLS. In summary, our results demonstrate that the transient editing system is a powerful tool to examine RBP binding to potential target RNAs under in vivo conditions. Variation of critical parameters, such as mutation of potential binding motifs in the RNA or co-expression of additional proteins and proteins can be easily achieved, contributing to our mechanistic understanding of RBP action in plants. Furthermore, we have developed a pipeline for a quantitative analysis of editing from Sanger sequencing results, making these studies fast, technically simple, and cost-effective compared to RNA-seq-based studies. Further advantages of our system are comparable expression levels of transgenes compared to analysing individual transgenic lines and that multiple design variants can be easily and rapidly screened. The PTB-hADARcd-NLS constructs established in this work can also be utilized to identify target RNAs in a transcriptome-wide manner via analysing stably transformed plants, as recently reported in hTRIBE studies of other plant RBPs (Arribas-Hernández et al. 2021a; Yin et al. 2023). In summary, hTRIBE-based analysis of RBP/RNA interactions is not only a powerful and sensitive approach to detect interactions in a global manner, but can also be used to functionally characterize individual binding events as demonstrated in our current study.

Previous studies establishing (h)TRIBE in *D. melanogaster* demonstrated that both ADARcd and hADARcd preserve some of the sequence and structure preferences at editing sites as known from the full-length ADAR proteins (McMahon et al. 2016; Xu et al. 2018). Accordingly, ADARcd and to a slightly reduced extent also hADARcd preferentially edited adenosine residues that are in the context of a 5’ uridine and a 3’ guanosine (Xu et al. 2018). The high confidence editing sites of OsDRB1-hADARcd also showed an overrepresentation of “UAG” (Yin et al. 2023). In our study, the major editing site detected in the *MS2* RNA (A43) fully matched this sequence context. In case of the *PTB2* pre-mRNA, the major editing site A374 was also preceded by a 5’ uridine, whereas the 3’ position was a cytidine. The consensus motifs “AAG” and “UAG” at editing sites were reported for ECT2/3-hADARcd (Arribas-Hernández et al. 2021a, b) and hADARcd-UBP1c (Zhou et al. 2021), with the “UAG” sequence context being enriched for the more strongly edited sites in case of ECT2-hADARcd. Accordingly, the hADARcd’s sequence preference can to some extent affect the editing outcome. Furthermore, transcriptome-wide studies in *D. melanogaster* revealed that (h)ADARcd preferentially edits bulged adenosine residues, i.e., the actual editing site should be unpaired but embedded within a double-stranded RNA region (McMahon et al. 2016; Xu et al. 2018). When comparing the editing extent and predicted structuredness for the *MS2-DsRED* RNA, we did not observe a correlation. However, as our targeted editing approach resulted in only relatively few editing sites compared to previous global profiling experiments, a correlation would be less likely to become visible.

We were also interested how the number of editing sites per target RNA and their position relative to the RBP binding site compared between our study and previous reports. The first study using TRIBE with ADARcd described for two of the three tested RBPs that in most cases one editing site was observed per target transcript, whereas multiple events were found in case of the third RBP (McMahon et al. 2016). The follow-up study including also a fusion with hADARcd demonstrated that usage of the hyperactive enzyme in general resulted in more editing sites per target compared to ADARcd. Actually, most positions were edited by both ADARcd versions, but in case of the less active ADARcd, editing was often below the significance threshold (Xu et al. 2018). Here in this work, we detected one major editing event for both types of RBP/RNA interactions. In case of the MS2 system, many additional significant events were identified, possibly also as a consequence of the more promiscuous RNA binding of the MS2 protein compared to PTBs. Furthermore, the interaction between MS2-hADARcd and the *MS2-DsRED* reporter RNA was expected to be much longer-lived compared to the association of the PTB2 fusion protein with the pre-mRNA in a splicing intermediate, thereby providing an extended time window for interaction and editing in case of the *MS2*-*DsRED *reporter RNA. The major editing site in the *MS2-DsRED* reporter was located at the beginning of the second copy of the two *MS2* loops, being in close vicinity of the two binding sites. The corresponding adenosine residue in the first *MS2* copy was not edited, despite having the identical sequence context. One possible explanation is that the editing event at A43 results from binding of MS2-hADARcd to the first MS2 loop, whereas upon its binding to the second loop no adenosine in an optimal position/context for efficient editing is available downstream. Interestingly, in case of the *DsRED-MS2* reporter also one major editing site was detected in the *MS2* region; however, this site was positioned at the end of the first *MS2* copy. The different editing positions in the MS2 region in comparison of the two reporter constructs suggests that the conformation of the *MS2* RNA module itself and/or the complex with the MS2 fusion protein differs depending on the *MS2* position relative to the *DsRED* sequence. An impact of the sequence and structural context on the editing outcome can also be deduced from the editing pattern of an *MS2*-containing RNA by MS2-hADARcd protein in rice protoplasts, showing highest editing percentages at two adenosine residues located in the opposite strands of a predicted base-paired region that is ~ 20 bp apart from one of the two *MS2* loops (Yin et al. 2023). In case of the *PTB2* pre-mRNA, the major editing event was located ~ 100 nt downstream of the pyrimidine-rich motifs required for PTB-dependent splicing and editing. In all of these cases, the major editing sites were in vicinity of the RBP binding region, which is in line with the findings from Xu et al. (2018) who reported for Hrp48-hADARcd that 40% and 32% of the editing sites, respectively, are within 100 nt and 100—500 nt relative to the binding position mapped by CLIP. In summary, whether an editing position is located up- or downstream of the RBP’s binding site probably depends primarily on the RNA structure and the positioning as well as orientation of the bound RBP-hTRIBE fusion protein on the target RNA.

### Functionality of (h)TRIBE fusion proteins

Ideally, (h)TRIBE fusion proteins should retain besides RNA binding also the other biological functions of the corresponding RBPs, as this could serve as an indicator whether the artificial fusions can faithfully recapitulate the authentic RBP behaviour. In this study, we have shown that hADARcd fusions of PTBs can trigger the same exon inclusion event that was previously reported to function in auto- and cross-regulation of PTB expression (Stauffer et al. 2010). Interestingly, the hTRIBE fusions preserved the specificity of PTB proteins, according to which the closely related PTB1 and PTB2, but not PTB3 from *A. thaliana* can induce the inclusion of a poison exon in the *PTB2* reporter mRNA. The correlation between the AS change and editing of the pre-mRNA, both of which did not occur in case of PTB3-hADARcd-NLS, suggested that PTB specificity is already determined at the level of target RNA recognition and binding, and not at later steps of AS regulation. Evidence that fusing hADARcd to RBPs does not disrupt their function was also provided by the observation that expressing ECT2-hADARcd under control of the *ECT2* promotor could almost fully rescue the phenotype of the triple mutant *ect2-1*/*ect3-1*/*ect4-2* (Arribas-Hernández et al. 2021a).

In conclusion, we have used the MS2 system and the interaction between plant PTB proteins and a specific target pre-mRNA to evaluate the potential of different editing enzymes in identifying in vivo targets of RBPs in plants in a functional context. Besides providing critical information and tools to design future (h)TRIBE studies for other plant RBPs, we have proven direct binding of the PTB2 fusion proteins to the *PTB2* pre-mRNA and gained novel insight into the mechanism and specificity of the corresponding AS event. Our work and the other aforementioned studies establishing (h)TRIBE and related approaches in animals and plants clearly demonstrate this technology’s enormous potential in deciphering RBP functions. Combined with techniques such as iCLIP for mapping exact binding positions of RBPs, the advantages of editing-based detection of in vivo RNA targets of RBPs, as in particular the high sensitivity and the potential to identify time-, cell- and isoform-specific RBP/RNA interactions, are expected to unravel completely novel aspects of RNA biology in plants and other organisms.

## Materials and methods

### Cloning procedures

All cds constructs and reporters were based on the vector pBinAR (Höfgen and Willmitzer 1992), which drives expression under control of the Cauliflower Mosaic Virus 35S promoter. Primers are listed in Supplementary Information, Table S1. All cds constructs include a Flag tag for immunological detection. PCR products and restriction reactions were cleaned-up using the GeneJET PCR Purification Kit (Thermo Fisher Scientific, USA). Insert sequences were confirmed using Sanger sequencing (Eurofins Genomics, Luxembourg).

Using overlap PCR, fusions of the *MS2* and *NLS-MS2* cds via the published linker (McMahon et al. 2016) with the sequence encoding the catalytic domain of ADAR from *Drosophila melanogaster* were generated. *MS2* and *NLS-MS2* cds were amplified from plasmid using JL19/JL20 and JL22/JL20 while attaching the linker relative to the 3’ end of the *MS2* cds. The ADARcd sequence was amplified with JL6/JL21 from plasmid SD06892 (http://flybase.org/reports/FBcl0278710.html) with the linker attaching to the 5’ end. The individual cds were fused using the outer primers with overlap PCR and cloned into pBinAR via *Kpn*I/*Bam*HI restriction sites. As a control, cds of ADARcd alone was amplified using JL4/JL5 and cloned into pBinAR via *Bam*HI/*Sal*I.

Introduction of the C to G point mutation into ADARcd resulting in E488Q for hyperactivity (Kuttan and Bass 2012; Xu et al. 2018) was achieved via PCR mutagenesis using JL29/JL30 on the original plasmid SD06892. The hADARcd, MS2-hADARcd and NLS-MS2-hADARcd constructs were cloned as described before for the corresponding ADARcd constructs.

The cds of *APOBEC1* from *Mus musculus* was fused to the cds of *MS2* via the same linker as for the ADARcd constructs. *APOBEC1* with the linker on the 5’ end and 3’ end was amplified from plasmid pNF-92 using JL32/JL33 and JL62/JL63, respectively. The MS2-linker sequence was amplified with JL31/JL20, while the linker-MS2 sequence was amplified with JL64/JL65. The different parts were fused using overlap PCR with the outer primers and cloned via *Bam*HI/*Xba*I into pBinAR.

The cds of *APOBEC3A* from *Homo sapiens* was amplified from plasmid HsCD00402611 (DNASU Plasmid repository, USA) and fused with the same linker as for the ADARcd constructs to the *MS2* cds. *APOBEC3A* was amplified using primers JL35/JL36, while *MS2* was amplified with JL19/JL20. To overcome APOBEC3A’s cytotoxicity in Agrobacteria, two different plant introns (Int_Cat_ and Int_PIV2*_) were introduced into the cds at two different positions, corresponding to intronic positions of the human *APOBEC3A* gene between exons 3 and 4 or exons 4 and 5. Int_Cat_ is a 190 nt long sequence derived from the castor bean catalase intron and was previously used to generate an intron-containing *Renilla reniformis* luciferase reporter gene (Cazzonelli and Velten 2003). Int_PIV2*_ is a 187 nt long intron (see also Supplementary Information, Fig. S3) derived from the 189 nt long Int_PIV2_ that has previously been successfully incorporated into a firefly reporter gene (Luke Mankin et al. 1997).

For introduction of the Int_Cat_ between exons 3 and 4, the 5’ part of the *MS2-APOBEC3A* cds was amplified with JL19/JL46, the Int_Cat_ sequence with JL46/JL48 and the 3’ part with JL49/JL36. To introduce the Int_Cat_ between exons 4 and 5, the 5’ part was amplified with JL19/JL50, the Int_Cat_ sequence with JL51/JL52, and the 3’ part with JL53/JL36. The Int_PIV2*_ sequence was inserted between exons 3 and 4 by amplifying the 5’ part of MS2-APOBEC3A with JL19/JL54, the Int_PIV2*_ with JL55/JL56, and the 3’ part with JL57/JL36. To insert the Int_PIV2*_ between exons 4 and 5, the 5’ part was amplified with JL19/JL58, the intron with JL59/JL60, and the 3’ part with JL61/JL36. Fusions were generated using overlap PCR with the outer primers JL19/JL36, followed by cloning into pBinAR via *Kpn*I/*Xba*I.

The reporter *MS2-DsRED* derived from annealing oligonucleotides ES3/ES4, which were cloned via an *Xma*I site into a *DsRED*-containing pBinAR plasmid. For *DsRED-MS2,* the oligonucleotides ES5/ES6 were annealed and cloned downstream of the *DsRED* cds via *Sal*I.

For PTB2-hADARcd constructs, the cds of At*PTB2* (*At5g53180*) was amplified using the primers JL1/JL3 while adding the linker as before. The cds of hADARcd with the linker was amplified with JL6/JL5. Subsequently, the primers JL1/JL5 were used for overlap PCR. The PTB2-hADARcd sequence was used as template for amplifying the SV40 NLS-PTB2-hADARcd (JL17/JL5) and PTB2-hADARcd-NLS bipartite (JL1/JL18) sequences. The *PTB2* cds alone was amplified with JL1/JL2. All four products were cloned via *Bam*HI/*Sal*I into pBinAR.

At*PTB1* cds was amplified from plasmid using the primers JL90/JL87 and fused to the hADARcd-NLS sequence (JL6/JL18) in an overlap PCR (JL90/JL18). Cloning into pBinAR was performed via *Bam*HI/*Sal*I. This plasmid was used as template for amplifying the NLS-PTB1-hADARcd sequence with the primers JL86/JL5. The resulting fragment was also cloned into pBinAR via *Bam*HI/*Sal*I. The At*PTB3* cds was amplified with the primers JL88/JL85 from a plasmid template. Fusion to the hADARcd-NLS sequence (JL6/JL18) was achieved by overlap PCR with the primers JL88/JL18, followed by cloning into pBinAR via *Bam*HI/*Sal*I. The NLS-PTB3-hADARcd sequence was amplified using the PTB3-hADARcd-NLS plasmid as template and the primers JL84/JL5. Cloning into pBinAR was also achieved via *Bam*HI/*Sal*I.

### Transient expression assay

*N. benthamiana* plants were grown on soil for 3 to 5 weeks under long day conditions (16 h light) at 20 to 24 °C and 30 to 60% relative humidity and the transient expression assay was performed as described in Wachter et al. (2007). *Agrobacterium tumefaciens* (C58C1) containing the respective constructs were grown over night in YEB medium (1 g/L yeast extract, 5 g/L beef extract, 5 g/L peptone, 5 g/L sucrose, 0.5 g MgSO_4_ × 7xH_2_O) at 28 °C. The cultures were centrifuged for 10 min at 4,000 g, the pellet resuspended in water and the OD_600_ adjusted to 0.8. Mixes of equal volumes were made containing the constructs for the silencing suppressor P19, a construct with an editing protein or control, the editing reporter and an infiltration control (in case of the *DsRED*-based reporters a *GFP* construct, and for the *GFP*-based reporter a *DsRED* construct). The third and fourth youngest expanded leaves of each plant were infiltrated with the mixes using a 1 mL syringe without needle. Material was harvested 2 to 5 days after infiltration, depending on the experiment.

### RNA isolation and reverse transcription

Around 100 mg of leaf material was harvested per sample. RNA was extracted using the UNIVERSAL RNA KIT (EURx, Poland), including an on-column DNase digestion for 15 min as described in the manual. The RNA was eluted in 40 µL RNase-free water. Reverse transcription from total RNA was performed using random hexamers (N_6_) and SuperScript^™^ II Reverse Transcriptase (Thermo Fisher Scientific, USA) following the supplier’s instructions. Furthermore, polyadenylated mRNA was reverse transcribed with AMV Reverse Transcriptase Native (EURx, Poland) and oligo dT primers according to the manual. For the time course experiment of MS2-hADARcd (Supplementary Information, Fig. S4), RNA and protein were extracted from the same samples, using the DNA+RNA+PROTEIN KIT from EURx (Poland), including an on-column DNase digestion for 15 min as described in the manual.

### PCR

PCR products for sequencing were amplified with S7 Fusion Polymerase^™^ (Mobidiag, Finland) according to the manufacturer’s instructions and using the oligonucleotides provided in Supplementary Information, Table S1. A homemade Taq polymerase was used with standard protocols for co-amplification PCRs of splicing patterns. All PCR products were separated on 2% or 3% agarose gels, stained with ethidium bromide, and visualized under UV light. Quantitative PCR for relative quantification of cDNAs transcripts was performed using the MESA BLUE qPCR MasterMix Plus (Eurogentec) according to manual and the CFX384 real-time PCR system (Bio-Rad). *GFP* was used as reference transcript for the samples from infiltrated *N. benthamiana* leaves.

### Quantitative analysis of editing frequencies via Sanger sequencing

RNA was isolated and reverse transcribed into cDNA as described before. The region of interest was PCR amplified using S7 Fusion Polymerase™ (Mobidiag, Finland). Subsequently, PCR products were purified with the GeneJET PCR Purification Kit (Thermo Fisher Scientific, USA), and subjected to Sanger sequencing using oligonucleotides (Supplementary Information, Table S1) complementary to the 5’ end of the PCR product and following the sequencing company’s instructions (Eurofins Genomics, Luxembourg). The chromatograms were quantified using DNADynamo version 1.556 (Blue Tractor Software Ltd). To test for adenosine to guanosine conversion ((h)ADARcd), for each adenosine position along the RNA sequence of interest, the areas of A and G peaks were determined and used for calculating the relative editing (i.e., G / (A+G)). For background correction, the relative editing determined for Luciferase or other types of control samples was subtracted. Subsequently, the following criteria were applied to define significant editing at each A position among the replicates: 1) the corrected editing value must be more than three times of its standard deviation above zero. 2) the uncorrected editing value must be at least twice the value of corresponding Luciferase control sample at this position. For testing APOBEC1 and APOBEC3A activity, cytosine-to-uracil conversion was determined using an equivalent approach. All editing analyses with calculations are included as Supplementary Information, Data S1.

### Protein extraction and immunoblot analysis

Around 100 mg plant material was used for total protein extraction with 300 µL extraction buffer (50 mM Tris–HCl, pH 7.5, 150 mM NaCl, 0.1% [v/v] Tween 20, 0.1% [v/v] β-mercaptoethanol, and Roche cOmplete™ ULTRA protease inhibitor cocktail). The extracts were centrifuged for 15 min at 4 °C and maximum speed. Protein concentrations were determined via Bradford and samples denatured for 5 min at 95 °C in 5 × SDS-sample buffer. If not stated otherwise, 20 µg total protein per sample was analysed using immunoblots. Protein samples from the time course experiment of MS2-hADARcd in Supplementary Information, Fig. S4 were extracted using the DNA+RNA+PROTEIN KIT from EURx (Poland), as described above. For SDS-PAGE and semidry immunoblotting, standard protocols were used. Detection was performed with the commercial antibodies α-FLAG from mouse (F3165, Sigma-Aldrich, USA), α-GFP from rabbit (A6455, Invitrogen, USA) and, for Fig. 3, α-GFP from mouse (11,814,460,001, Roche, Switzerland). When included as loading control, Ponceau S staining was performed after blotting and before immunodetection.

### CAPS assay

For the Cleaved Amplified Polymorphic Sequences (CAPS) assay, cDNA generated by reverse transcription with random hexamer primers was used in a first PCR with primers P2/P3. The resulting PCR product was used for a second PCR with primers P2/JL108 introducing two base pair mutations that generate in case of editing at A374 a *Pst*I site (see Supplementary Information, Fig. S8 for a graphical depiction). Both PCR reactions were performed using a homemade Taq polymerase with standard protocols. The products from the second PCR were digested with *Pst*I-HF (NEB) for 2 h at 37 °C, followed by separation on 3% agarose gels, staining with ethidium bromide, and visualization using UV light. For a quantitative analysis, amounts of uncleaved and cleaved products were determined using the 2100 Bioanalyzer and the DNA1000 kit (Agilent Technologies, Santa Clara, CA, USA).

### Validation of assay for peak ratio analysis

The reporter *MS2-DsRED* was amplified with a homemade Taq polymerase (primers JL44/JL45) following standard protocols and products ligated into pGEM-T (Promega, USA). A mutagenesis PCR was performed to introduce an A-to-G mutation at position P499 using the primers JL42/JL43 (with position P1 defined as the first nucleotide at the 5’ end of *MS2-DsRED*). S7 Fusion Polymerase™ (Mobidiag, Finland) was used according to its manual. For plasmid amplification, the constructs were transformed into *Escherichia coli* (XL1-Blue). Upon overnight growth of the *E. coli* cultures in LB medium at 37 °C, plasmids were purified with the GeneJET^™^ plasmid miniprep kit (Thermo Fisher Scientific, USA). Sanger sequencing revealed an additional mutation of C to T at position P193, which was used as additional reference. For peak height comparison, the mutated and non-mutated reporters were amplified with S7 Fusion Polymerase^™^ (Mobidiag, Finland) using the primers JL26/AW62. Then purified PCR products were mixed in different ratios according to Supplementary Information, Fig. S1 and sent for Sanger sequencing. Peak ratios at positions 193 and 499 were quantified using DNADynamo version 1.556 (Blue Tractor Software Ltd).

### Accession numbers

At3g01150 (PTB1), At5g53180 (PTB2), At1g43190 (PTB3).

### Supplementary Information

Below is the link to the electronic supplementary material.

Supplementary file1 (XLSX 4458 KB)

Supplementary file2 (XLSX 19 KB)

Supplementary file3 (PDF 1459 KB)

## Data Availability

All relevant data are included in the main section or the Supporting information of the article.
