# Supplementary material for: A transient in planta editing assay identifies specific binding of the splicing regulator PTB as a prerequisite for cassette exon inclusion
Source: Plant Mol Biol. 2024 Mar 5;114(2):22. doi: 10.1007/s11103-024-01414-3 (PMC10914923; doi:10.1007/s11103-024-01414-3)
Supplement: Supplementary file 3 — Supplementary file3 (PDF 1459 KB) [file 11103_2024_1414_MOESM3_ESM.pdf]

Supplementary Information

A transient *in planta* editing assay identifies specific binding of the splicing regulator PTB as a prerequisite for cassette exon inclusion

Jorinde Loeser, Julia Bauer, Kim Janßen, Kevin Rockenbach, and Andreas Wachter*

Institute for Molecular Physiology (imP), University of Mainz, Hanns-Dieter-Hüsch-Weg 17, 55128 Mainz, Germany

*Corresponding author: wachter@uni-mainz.de, phone: +49 6131 39-28908

(A)

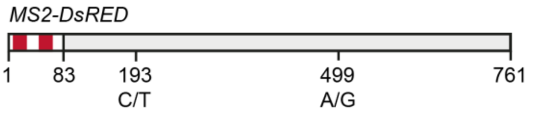

(B)

		Ratio (wt:mutant)	1:0	19:1	9:1	4:1	1:4	1:9	1:19	0:1
		Expected [%]	0	5	10	20	80	90	95	100
Measured [%]	mean	**C>T(193)**	3	5	9	15	82	92	94	99
		**A>G(499)**	2	4	5	11	52	67	62	68
	stdev	**C>T(193)**	2.1	2.9	3.6	9.2	7.1	2.7	2.5	0.1
		**A>G(499)**	0.4	1.7	4.1	6.7	16.5	11.2	9.9	12.5

(C)

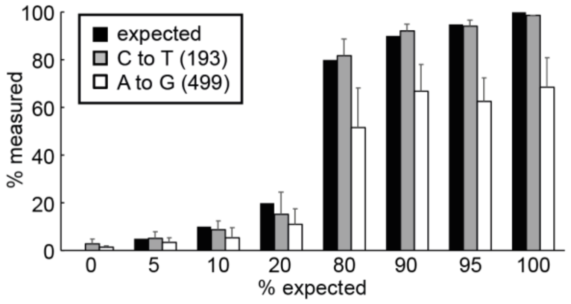

Fig. S1 Quantification of peak heights from Sanger sequencing can be used as editing readout. **(A)** Cartoon of the *MS2-DsRED* reporter drawn to scale with point mutations introduced in corresponding plasmids at positions 193 (C to T) and 499 (A to G), with position 1 defined as the first nucleotide at the 5' end of *MS2-DsRED*. **(B, C)** Quantitative analysis of peak heights from Sanger sequencing upon mixing plasmids containing the WT version of *MS2-DsRED* and *MS2-DsRED* with the two point mutations (C193T, A499G) at indicated ratios. Expected and measured signal percentages of the mutated bases are displayed as numbers (B) and in a bar graph (C). Average and stdev are based on 2 - 4 independent replicates.

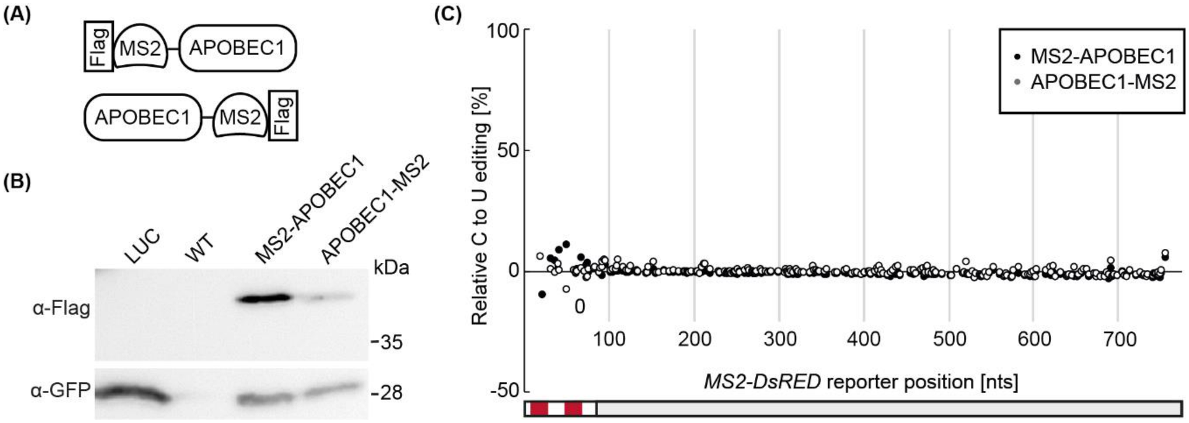

Fig. S2 No significant editing is detected for APOBEC1 fused to MS2. **(A)** Cartoons of the constructs for expressing fusions of APOBEC1 with MS2. **(B)** Immunoblot analysis of samples from *Nicotiana benthamiana* leaves transiently transformed with MS2-APOBEC1 and APOBEC1-MS2. Leaves transformed with Luciferase construct (LUC) and untransformed wild type (WT) serve as controls. GFP from co-transformation was detected as loading control. Samples were taken 3 days after infiltration and 20 µg total protein was loaded per lane. Positions of relevant size marker bands are indicated. **(C)** Quantitative analysis of C to U editing for all C bases along the *MS2-DsRED* reporter. Samples were taken three days after infiltration and editing determined via Sanger sequencing of RT-PCR products. Data based on 5 (MS2-APOBEC1) and 10 (APOBEC1-MS2) biological replicates derived from 6 independent experiments, respectively. No significant editing is detected according to the criteria described in the methods section.

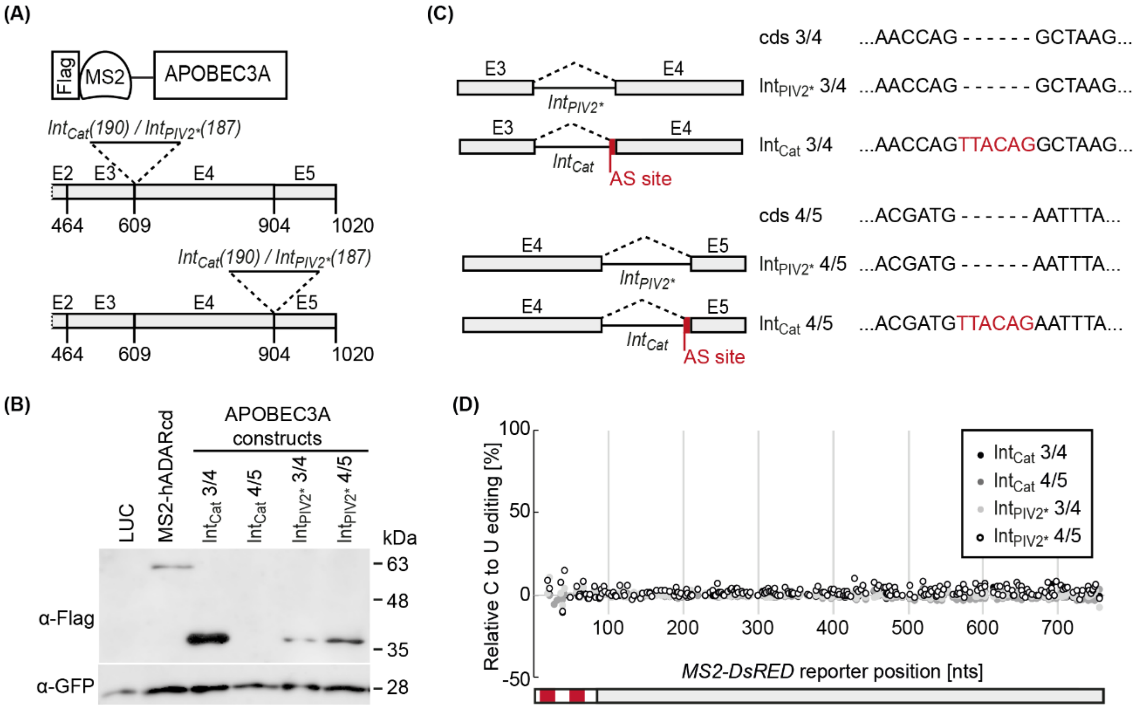

(E)

```
IntPIV2   1   GTAAGTTTCTGCTTCTACCTTTGatatatatataataattatcattaattagtagtaata  60
              ||||||||||||||||||||||  ||||||||||||||||||||||||||||||||||||
IntPIV2*  1   GTAAGTTTCTGCTTCTACCTTTG--ATATATATAATAATTATCATTAATTAGTAGTAATA  58

IntPIV2   61  taatatttcaaatatttttttCAAAATAAAAGAATGTAGTATATAGCAATTGCTTTTCTG  120
              ||||||||||||||||||||||||||||||||||||||||||||||||||||||||||||
IntPIV2*  59  TAATATTTCAAATATTTTTTTCAAAATAAAAGAATGTAGTATATAGCAATTGCTTTTCTG  118

IntPIV2   121 TAGTTTATAAGTGTGTATATTTTAATTTATAACTTTTCTAATATATGACCAAAATTTGTT  180
              ||||||||||||||||||||||||||||||||||||||||||||||||||||||||||||
IntPIV2*  119 TAGTTTATAAGTGTGTATATTTTAATTTATAACTTTTCTAATATATGACCAAAATTTGTT  178

IntPIV2   181 GATGTGCAG  189
              |||||||||
IntPIV2*  179 GATGTGCAG  187
```

Fig. S3 No significant editing is detected for APOBEC3A fused to MS2. **(A)** Cartoon of the MS2-APOBEC3A fusion construct (top), and insertion positions of two plant introns (Int_{Cat}, 190 nts; Int_{PIV2*}, 187 nts) between the third and fourth (middle) or fourth and fifth exon (bottom) of the APOBEC3A cds. Middle and bottom cartoons drawn to scale. Numbers below cartoons indicate nucleotide positions of the APOBEC3A exons without the plant introns but in the context of the fusion construct, i.e., including the sequences for the Flag tag and MS2. Note that Int_{PIV2*} is two nucleotides shorter than the originally published Int_{PIV2} sequence (see also below in (E)). **(B)** Immunoblot analysis of samples from *Nicotiana benthamiana* leaves transiently transformed with MS2-APOBEC3A constructs containing one of the two different introns between the third and fourth (Int_{Cat} 3/4; Int_{PIV2*} 3/4) or fourth and fifth (Int_{Cat} 4/5; Int_{PIV2*} 4/5) exon. Leaves transformed with luciferase (LUC) and MS2-hADARcd construct serve as controls. GFP from co-transformation was detected as loading control. Samples were taken 3 days after infiltration and 20 µg total protein was loaded per lane. **(C)** Cartoons of intron splicing upon expression in *Nicotiana benthamiana,* with an alternative 3' splice site (AS site) choice in case of the Int_{Cat}, resulting in an insertion of 6 nts (TTACAG), depicted in red, and correct splicing of Int_{PIV2*}. This insertion results in proteins with two additional amino acids: leucine and glutamine (exon border 3/4) or valine and threonine (exon border 4/5). **(D)** Quantitative analysis of C to U editing for all C bases along the *MS2-DsRED* reporter, co-expressed with the MS2-APOBEC3A constructs containing the intron sequences at the indicated positions. Samples were taken three days after infiltration and editing determined via Sanger sequencing of RT-PCR products. 4 biological replicates derived from two independent experiments. No significant editing is detected according to the criteria described in the methods section. **(E)** Sequence alignment of the original Int_{PIV2} (Luke Mankin et al. 1997) and the shortened version Int_{PIV2*} used in this study, lacking one "AT" dinucleotide in a repetitive region due to a cloning artifact. The splicing analysis indicated that this deletion does not affect correct intron removal.

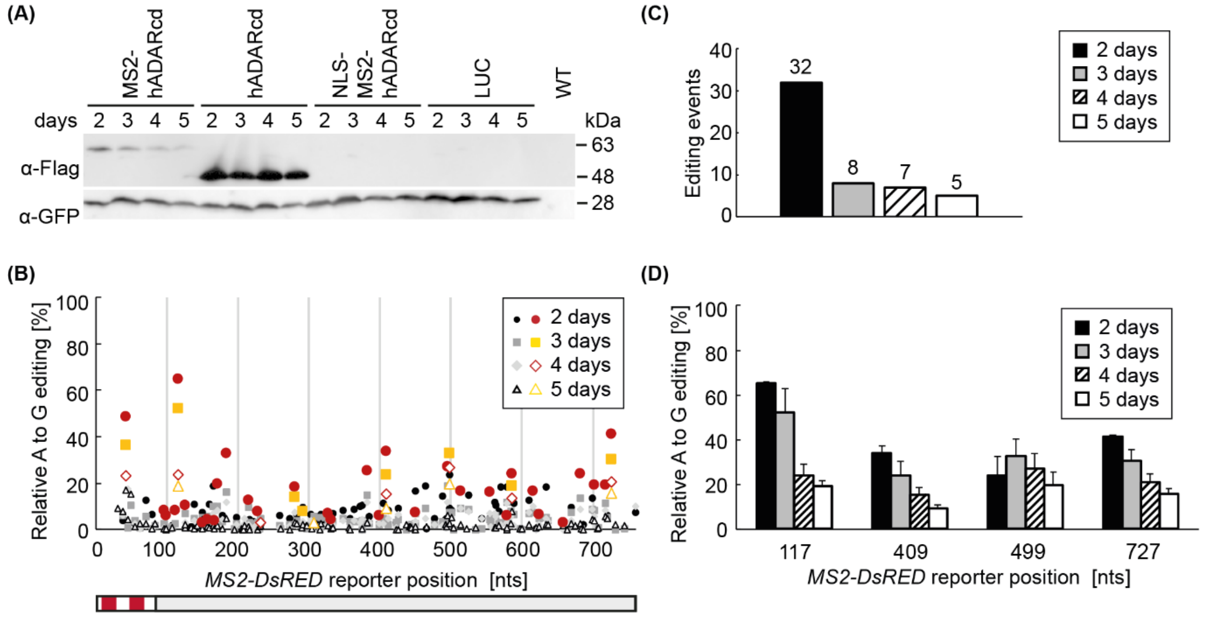

Fig. S4 Time course of editing mediated by MS2-hADARcd. **(A)** Immunoblot analysis of samples from *Nicotiana benthamiana* leaves transiently transformed with MS2-hADARcd, hADARcd and NLS-MS2-hADARcd and harvested over 2 - 5 days after infiltration. Leaves transformed with Luciferase construct (LUC) and untransformed wild type (WT) serve as controls. GFP from co-transformation served as loading control. Protein and RNA were extracted from the same samples and 20 µL protein extract (from total volume of 100 µL derived from 5 leaf discs) was loaded per lane. **(B)** Quantitative analysis of A to G editing for all A bases along the *MS2-DsRED* reporter, co-expressed with MS2-hADARcd in a transient expression assay at indicated time points. 3 - 6 biological replicates derived from 3 independent experiments, analysed by Sanger sequencing. Significant events highlighted in colour. **(C, D)** Number of significant editing events from the quantitative analysis in (B) compared between the days (C) and the extent of editing depicted for four positions (D), along with standard deviation of the corrected editing quantity.

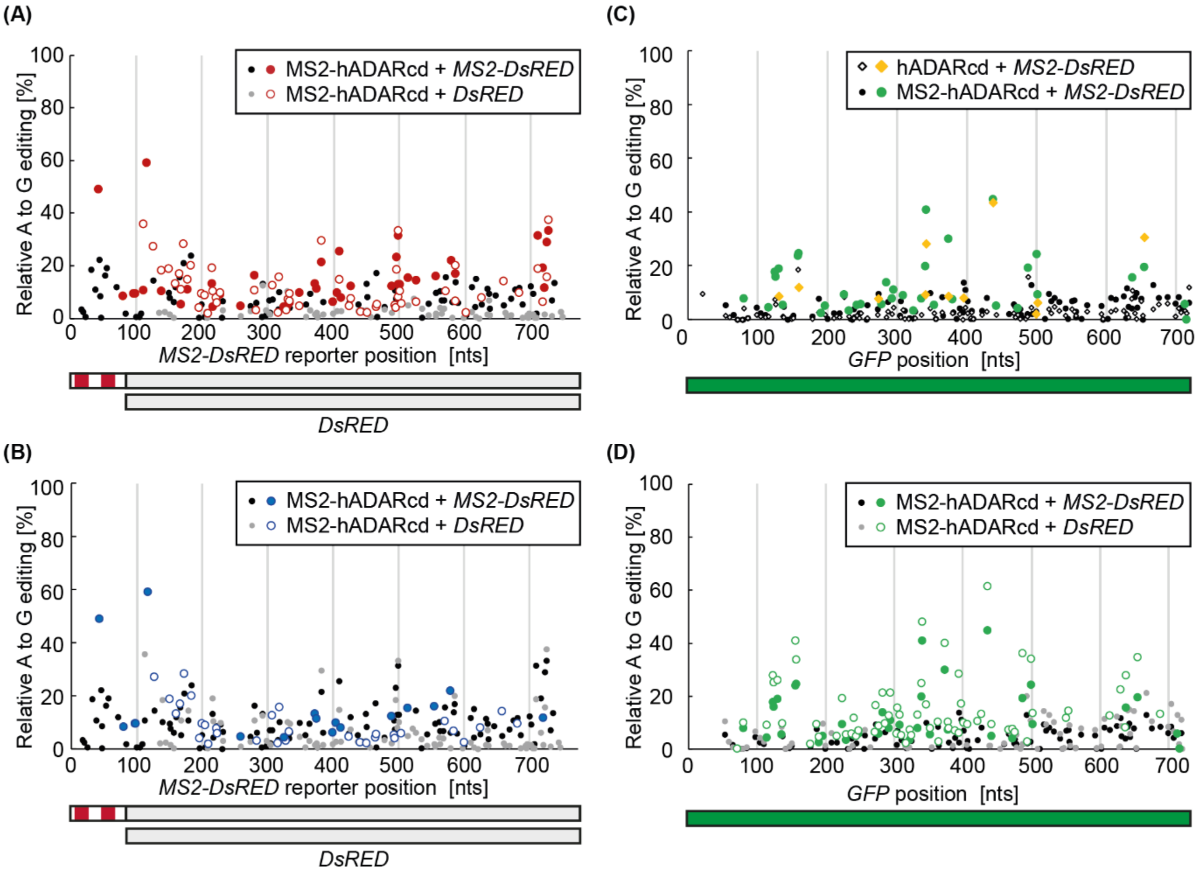

Fig. S5 Editing by MS2-hADARcd is not restricted to *MS2*-containing RNAs. **(A, B)** Quantitative analysis of A to G editing for the A bases along *MS2-DsRED* or *DsRED* RNA with significant changes in red (A) or with significant and unique events depicted in blue (B). **(C)** Quantitative analysis of A to G editing by MS2-hADARcd or hADARcd for all A bases along *GFP* RNA co-expressed with *MS2-DsRED*. Significant events are highlighted in colour. **(D)** Quantitative analysis of A to G editing of MS2-hADARcd for all A bases along *GFP* RNA co-expressed with either *MS2-DsRED* or *DsRED*. Significant events are highlighted in green. Data for all four diagrams derived from Sanger sequencing of four biological replicates each; transient expression for 3 days in *N. benthamiana*.

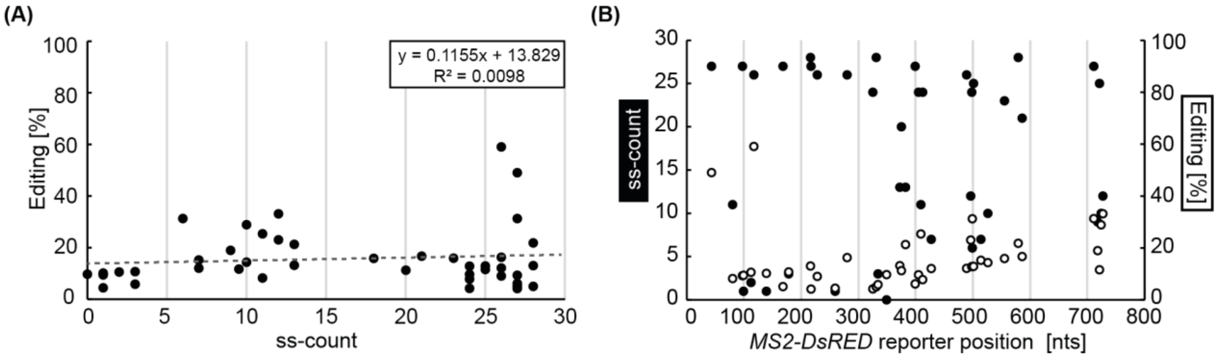

Fig. S6 Extent of editing is not correlated with RNA structure prediction for the *MS2-DsRED* reporter. **(A)** Relative editing plotted versus the probability of single-strandedness (ss-count, defined via the mfold web server, http://www.unafold.org) for the nucleotides that were significantly edited by MS2-hADARcd. Editing percentage based on four biological replicates. A regression line (grey) and its equation and R^2 are depicted. **(B)** Single-strandedness (filled dots) and editing percentages (open dots) of the same events as in (A), but displayed along the *MS2-DsRED* reporter sequence.

(A)

...**GT**AAGTGGATCACACACCTAATTGGTTTCGTTAGATGATGTAAATAATAAATATATTGCTAAAAGATGTTTTGGAGA
AATGCCGATGATTAGGTTGTAGGTTGTCGTGCTTTTGAGAATTTTCTTTTGACGTGAATGTAAATGGATCAGC**AG**GGC
CATGTCAAGGCAATATCTTCAGTGGCAGCCTAATGCATTAGGAGAAAGAGCACATGTGTGTTTGTTTCCTTTCTCCTT
TCCCTGACACTGCCTCTCTTTTCCCTACTCTCTCCCTCACACTTCCCTTTATCAGATATATATTCTAGGATTTGTGAG
ATATGGTCTTACATAGGTGTAGTTAGTGTTTTTGTTTGATATCATAGGCAGGGGCCAGCTCTACCTTACATAGGTGTA
GTTAGTATTTTTGTTTGATATGATAGGATGCTACTCTATCTGTATCTGCACATCTTTTGTGATATAGGACTGTGTCAT
AGTTTATGTGACTGATGAATCTATCTAGAGGTTCATCAATCTGCTAACCTTTTGGGCTGTTCCTTGTGTCTAATGTGA
TTGTTTGATATGTTGGAAGACAAATCTTATGCCTTCCAGAGAGCGTAACCTGAAATCAAGCTTGTCACGTCATATTAT
GTTGGTTTAATTTGCTATCTTAACTTTTGTCATGCTGTATGAATTTTATACTTAGAGAGTTGGCATTAACTAAAGTTT
TCTTGGAATGTTGCTGAAGGGCAAAATAATTACAGATGCTACACTGGAGTCTTTATAGTTAACTCTTCTGGCTCTTTT
TTTCTTTGTTGTTCTAATTGCTGGTTTGCCATGC**AG**...

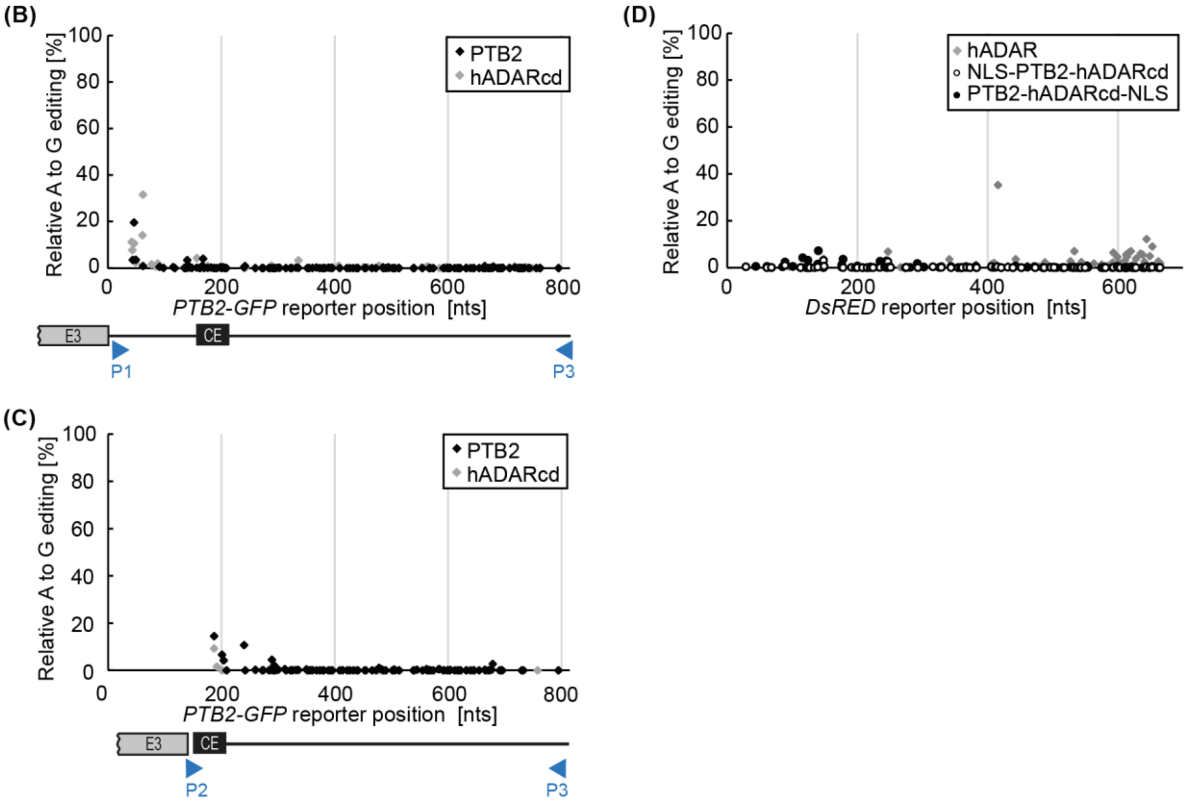

Fig. S7 No background editing is detected for the *PTB2-GFP* reporter and *DsRED*. **(A)** Sequence of *PTB2-GFP* reporter for the region from the start of the intron upstream of the cassette exon (in blue) to the end of the intron downstream of it. Constitutive and alternative splicing sites highlighted with green and yellow shading, respectively. Pyrimidine-rich motifs with grey shading were changed to purines (C to G; T to A) in the mutant reporter. **(B, C)** Quantitative analysis of A to G editing frequencies for the A bases along the region flanking the cassette exon of the reporter, transiently transformed with PTB2 or hADARcd. Two intermediate splice versions were sequenced with one (B) containing both intronic regions surrounding the cassette exon (P1/P3 primer combination) and the other (C) having the first intron already spliced out (P2/P3 primer combination). Aligned reporter region indicate approximate positions of editing in intron/exon context. Samples were taken three days after infiltration and editing determined via Sanger sequencing of RT-PCR products. Data derived from 3-6 biological replicates. No significant editing events were detected. **(D)** Quantitative analysis of A to G editing frequencies for the A bases along *DsRED*, co-expressed with the *PTB2-GFP* reporter and hADARcd, NLS-PTB2-hADARcd-NLS and PTB2-hADARcd-NLS in *N. benthamiana*. Samples were taken three days after infiltration and editing determined via Sanger sequencing of RT-PCR products. Data derived from 6 biological replicates. No significant editing events were detected.

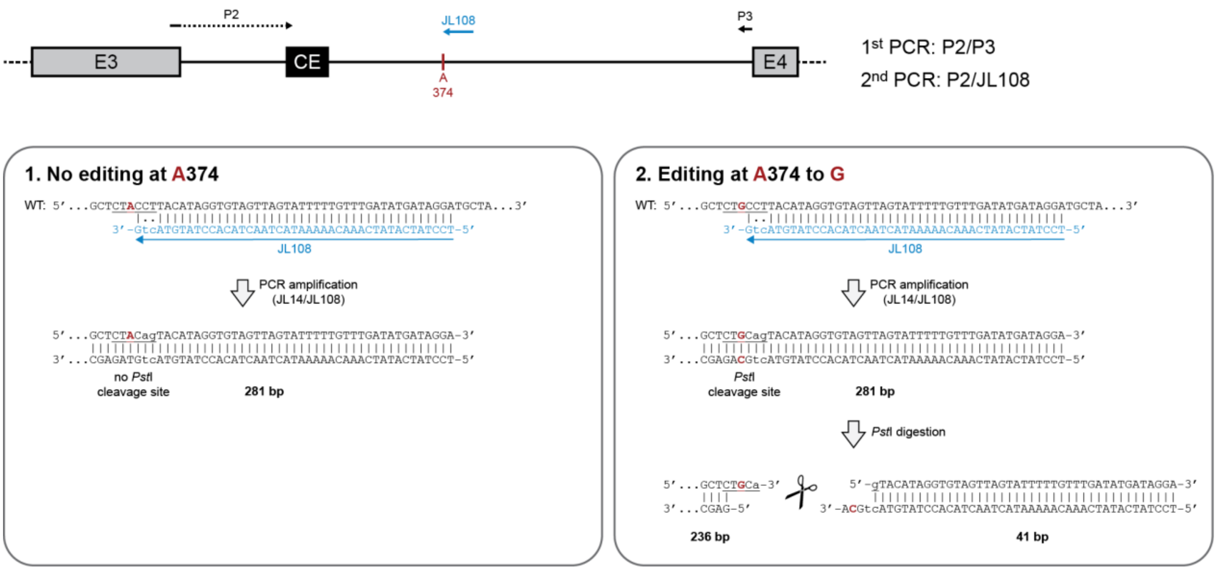

Fig. S8 Strategy of CAPS-based detection of A-to-G editing at position A374. Upper cartoon shows A374 position in the context of the *PTB2* pre-mRNA. Boxes and lines depict exons and introns, respectively; CE, cassette exon. Only region from exon 3 to exon 4 is displayed. Arrows depict primer binding positions, with P2 spanning an exon-exon border as indicated by dotted line. Two subsequent PCR reactions were performed: 1) from precursor cDNA with primers P2/P3; 2) product from first PCR served as template for second PCR with primers P2/JL108. Lower part illustrates CAPS procedure, with primer JL108 introducing two mismatches to complement a *Pst*I restriction site in case of A-to-G editing at position A374. In the absence of editing, no *Pst*I site is generated and a fragment of 281 bp corresponding to the full-length PCR product can be detected (left). In case of editing at A374, *Pst*I cleavage of the PCR product results in the release of a 41 bp fragment and detection of a 236 bp product (right); these sizes only refer to the double-stranded regions and do not include the 4-nts-long single strand overhang present in both fragments upon digestion. Underlined nucleotides correspond to the sequence that can be transformed into a *Pst*I site (CTGCAG) by PCR mutagenesis with JL108 in case editing of the RNA had occurred.

Reference

Luke Mankin, S.; Allen, George C.; Thompson, William F. (1997): Introduction of a plant intron into the luciferase gene of Photinus pyralis. In Plant Molecular Biology Reporter 15 (2), pp. 186–196. DOI: 10.1007/BF02812270.
